# Immunolabeling-compatible PEGASOS tissue clearing for high-resolution whole mouse brain imaging

**DOI:** 10.3389/fncir.2024.1345692

**Published:** 2024-04-17

**Authors:** Pan Gao, Matthew Rivera, Xiaoxiao Lin, Todd C. Holmes, Hu Zhao, Xiangmin Xu

**Affiliations:** ^1^Department of Anatomy and Neurobiology, School of Medicine, University of California, Irvine, Irvine, CA, United States; ^2^Department of Physiology and Biophysics, School of Medicine, University of California, Irvine, Irvine, CA, United States; ^3^Center for Neural Circuit Mapping, University of California, Irvine, Irvine, CA, United States; ^4^Chinese Institute for Brain Research, Beijing, China

**Keywords:** tissue clearing, whole-mount immunostaining, circuit tracing, Alzheimer’s disease, Light-Sheet

## Abstract

Novel brain clearing methods revolutionize imaging by increasing visualization throughout the brain at high resolution. However, combining the standard tool of immunostaining targets of interest with clearing methods has lagged behind. We integrate whole-mount immunostaining with PEGASOS tissue clearing, referred to as iPEGASOS (immunostaining-compatible PEGASOS), to address the challenge of signal quenching during clearing processes. iPEGASOS effectively enhances molecular-genetically targeted fluorescent signals that are otherwise compromised during conventional clearing procedures. Additionally, we demonstrate the utility of iPEGASOS for visualizing neurochemical markers or viral labels to augment visualization that transgenic mouse lines cannot provide. Our study encompasses three distinct applications, each showcasing the versatility and efficacy of this approach. We employ whole-mount immunostaining to enhance molecular signals in transgenic reporter mouse lines to visualize the whole-brain spatial distribution of specific cellular populations. We also significantly improve the visualization of neural circuit connections by enhancing signals from viral tracers injected into the brain. Last, we show immunostaining without genetic markers to selectively label beta-amyloid deposits in a mouse model of Alzheimer’s disease, facilitating the comprehensive whole-brain study of pathological features.

## 1 Introduction

Classical histological approaches that rely on brain sectioning have been a foundational technology for anatomical neuroscience research for over 100 years. To better understand the structural features of healthy or diseased brains, visual interrogation in three-dimensions is imperative. However, cellular resolution volume imaging is difficult due to tissue opacity and limited light penetration into deep brain samples. To circumvent these optical limitations, scientists mechanically section 2D brain slices and digitally reconstruct imaged slices to artificially render a 3D view of the whole brain (Stille et al., 2013). This approach has inherent problems with registration between sections and physical damage at the surface of tissue sections, thus impeding the accuracy of high-resolution reconstruction. Furthermore, the multiple steps of mechanical/physical brain sectioning, mounting, processing, and scanning individual slices are labor-intensive and error-prone. The first attempt at tissue clearing is a century old (Spalteholz, 1914). There is renewed interest in tissue clearing methods driven by technical imaging improvements, including Light-Sheet Microscopy (Dodt et al., 2007) that allows single-cell resolution optical scanning of large samples such as entire mouse brains. In Light-Sheet Microscopy, a thin sheet of laser light is directed into the sample from the side. This light sheet selectively excites fluorophores within the illuminated plane. The fluorescence emitted by the excited fluorophores is captured by a camera or a detector placed perpendicular to the light sheet. This configuration ensures that only the fluorescence generated within the thin sheet of illumination is detected, reducing out-of-focus light and improving image contrast. Multiple fields of view (FOV) at the same plane are then overlaid together to generate a single *“optical plane” or “optical section”*. These optical sections can be acquired at different depths by adjusting the position of the light sheet and the detection plane. These multiple optical sections are then used to generate a three-dimensional reconstruction of the whole tissue, providing detailed insights into the spatial organization of structures within the sample. This approach allows fast and high-resolution imaging of large, cleared tissues or organs.

There has been a surge of improved tissue clearing protocols (Hama et al., 2011; Chung et al., 2013; Renier et al., 2014; Susaki et al., 2014; Jing et al., 2018; Kosmidis et al., 2021). Although different chemicals are used in these protocols, the underlying physical principle of tissue clearing is similar. They include steps of tissue fixation, decalcification to remove calcium phosphate when the specimen includes bones, decolorization to remove endogenous pigments, delipidation to remove lipids, and refractive index (RI) matching to calibrate the refractive index of specimen and imaging medium to maximize transparency. Chemically, current tissue clearing techniques can be categorized into two major approaches based on the final RI matching medium used: hydrophobic and hydrophilic tissue clearing (Ueda et al., 2020). These are also known as organic solvent-based and aqueous-based tissue clearing, respectively. Hydrophobic tissue clearing confers an advantage by providing fast clearing kinetics due to the higher diffusion rate of small molecules (Dodt et al., 2007; Renier et al., 2014; Jing et al., 2018). Additionally, it can preserve specimens for up to a year for multiple imaging sessions and reanalysis based on the dehydration step that hardens the sample. However, the dehydration step tends to quench fluorescent signals and shrinks tissue size. In contrast, hydrophilic tissue clearing uses water-soluble reagents for the final RI matching step (Hama et al., 2011; Ke et al., 2013; Susaki et al., 2014). The transparency quality of hydrophilic tissue clearing is optically inferior compared to hydrophobic tissue clearing, but it has advantages in biosafety and fluorescence signal protection.

Fluorescent reporters are frequently used to visualize molecules of interest. Most currently available clearing protocols have been developed to visualize fluorescent reporter proteins delivered through combined applications of transgenic reporter lines and virus-assisted reporter gene delivery. However, the fluorescent signal intensity based on genetically targeted methods typically decreases throughout the clearing process. Furthermore, in some cases, the reporter line or virus-assisted reporter gene yields inherently weak expression, the fluorescent signal may become indistinguishable from the background noise post-clearing. Therefore, to further extend imaging capability, clearing methods need to be compatible with immunolabeling techniques. However, insufficient antibody penetration into deeper parts of large tissues, such as the whole brains, limits whole-brain labeling. To overcome this limitation, we combine whole-mount immunostaining and tissue clearing protocols from two well developed protocols: iDISCO staining (Renier et al., 2014) + PEGASOS clearing (Jing et al., 2018). We call our updated hybrid method iPEGASOS. iPEGASOS yields superior transparency while preserving strong fluorescence signals over a year and enables deep antibody penetration in large-sized tissues, including whole mouse brain. To demonstrate the efficacy and versatility of iPEGASOS, we present brain-wide mapping of cellular resolution labels in 1) transgenic reporter mouse brains (DAT-Cre; Ai9 and VIP-Cre; Ai9), 2) mouse brains with viral tracer injection, and 3) amyloid beta-immunostaining in Alzheimer’s disease mouse model brains (5xFAD mice). The synergistic application of iPEGASOS and Light-Sheet Microscopy enables profiling of cell types throughout the entire brain, high-resolution interrogation of neural circuits down to the single-axon level, and investigation of the temporal progression of Alzheimer’s disease-related pathological features, such as amyloid plaque deposition, in 3D within whole mouse brains.

## 2 Methods

### 2.1 Animals

Adult mice (more than 8 weeks), both males and females, with genotypes including VIP-Cre;Ai9, DAT-cre;Ai9, C57BL/6, Tie2-GFP, APP knock in, 5xFAD, 3xTg-AD, were used in the experiments. All experiments were conducted according to the National Institutes of Health guidelines for animal care and use and were approved by the Institutional Animal Care and Use Committee and the Institutional Biosafety Committee of the University of California, Irvine.

### 2.2 Perfusion and tissue preparation

The mice were transcardially perfused. First, 50–100 mL ice-cold PBS (NaCl: 137 mM, KCl: 2.7 mM, Na_2_HPO_4_: 10 mM, KH_2_PO_4_: 1.8 mM) was injected transcardially to wash out the blood. Then 50 mL 4% paraformaldehyde (PFA) in PBS was infused for fixation. The brains were dissected out and postfixed in 4% PFA for 12 to 24 h in 4°C, then transferred to PBS.

### 2.3 Preparation of iPEGASOS solutions

Solutions were adopted from the PEGASOS (Jing et al., 2018) and iDISCO whole-mount Labeling protocol (Renier et al., 2014).

#### 2.3.1 Decolorization solution

Quadrol (N,N,N′,N′-Tetrakis(2-hydroxypropyl) ethylenediaminel) (Sigma-Aldrich 122,262) was diluted with distilled H_2_O to a final concentration of 25% v/v.

#### 2.3.2 Gradient tB delipidation solution

Pure tert-Butanol (tB) (Sigma-Aldrich 360,538) was diluted with distilled H_2_O to prepare gradient delipidation solutions at 30% v/v, 50% v/v and 70% v/v. Quadrol was then added with 3 ~ 5% w/v final concentration to adjust the pH to above 9.5.

#### 2.3.3 Staining pretreatment solution

PBS/0.2% Triton X-100(PTX.2) was composed of 0.2% Triton X-100 in PBS.

#### 2.3.4 Permeabilization solution

Composed of 0.2% Triton X-100, 20% DMSO, 0.3 M glycine in PBS.

#### 2.3.5 Blocking solution

Composed of 0.2% Triton X-100, 10% DMSO, 3% normal donkey serum in PBS.

#### 2.3.6 Staining solutions

PBS/0.2% Tween-20 with 10 μg/mL heparin (PTwH) was composed of 0.2% Tween-20 with 10 μg/mL heparin in PBS. Primary antibody diluted in PTwH with 5% DMSO, 1% Donkey Serum depends on recommended dilution ratio. Secondary antibody diluted in PTwH with 1% Donkey Serum depends on recommended dilution ratio. Centrifuging antibody solution at 20000 g for 10 min can prevent formation of precipitates in the sample. Alternatively, syringe-filter the solution at 0.2 μm.

#### 2.3.7 tB-PEG dehydration solution

Dehydrating solution was composed of 70% v/v tert-Butanol, 25 ~ 27% v/v Poly(ethylene glycol) methacrylate Mn 500 (PEGMMA500) and 3 ~ 5% w/v Quadrol.

#### 2.3.8 BB-PEG clearing medium (refractive index R.I. 1.543) (also Light-Sheet Microscope imaging medium)

BB-PEG was prepared from mixing 75% v/v benzyl benzoate (BB, Sigma-Aldrich W213802) and 20 ~ 22% v/v PEGMMA500 supplemented with 3 ~ 5% w/v Quadrol together. The fresh medium was a colorless liquid with low viscosity and turned slightly yellow in a week.

### 2.4 iPEGASOS passive immersion procedure for whole mouse brains or hemispheres

We provide a step-by-step protocol of iPEGASOS in the Supplementary material.

The clearing and staining procedures were mentioned previously (Renier et al., 2014; Jing et al., 2018). The whole process was performed on a shaker at 37°C. Overnight or 24 h postfixed brain tissues were washed in PBS twice for 1 h each time. Samples were then immersed into *decolorization solution* for 2 days with daily change. Following that, samples were placed in gradient *tB delipidization solution* for 2 days. 30% tB for ~4 h, 50% tB for ~6 h and 70% tB for the rest of time.

Samples were then washed in PBS for 1 h twice and *PTX.2* 1 h twice, followed by *permeabilization solution* for 2 days. After that, the pretreated samples were immersed in *blocking solution* for another 2 days, then washed in *PTwH* 1 h twice and incubated in staining solution based on recommended dilution ratio for 5 days or longer (based on sample size). Samples were then washed in PTwH for 5 times per day for 2 days before switching to secondary antibody solutions for 5 days or longer (based on sample size). Samples were finally washed in PTwH for 5 times per day for 2 days.

Following the final wash with PTwH, the second round of gradient tB delipidation was performed on the samples using 30, 50 and 70% v/v tB for 2 days. Later samples were incubated in tB-PEG-MEM dehydration solution for 1 to 2 days with daily change. Samples were then switched to new containers with clearing medium BB-PEG for 2 days. Then the samples were imaged under the Light-Sheet Microscope. After imaging, the samples can be stored in clearing medium at room temperature for at least a year. We have samples stored for over 2 years without significant signal loss.

### 2.5 PEGASOS passive immersion procedure for mouse brain or hemispheres

Similar to iPEGASOS, only remove first round of delipidation and immunostaining steps.

### 2.6 iPEGASOS and PEGASOS incubation duration for different size samples

**Table 1:** 

Chemicals and steps		Whole brain or Half brain	30 μm mouse brain slices	Incubation condition
Decolorization	25% Quadrol	2 days with daily change	1 h	37°C in shaker
First round of Delipidation (no needed for PEGASOS)	30% tB + 3% Quadrol	4 h	1 h	
	50% tB + 3% Quadrol	6 h	1 h	
	70% tB + 3% Quadrol	1 day	1 h	
Immunostaining (no needed for PEGASOS)	iDISCO based solution	~14 days	2 days (same as iDISCO protocol for 30 μm mouse brain slices)	
Second round of Delipidation	30% tB + 3% Quadrol	4 h	1 h	
	50% tB + 3% Quadrol	6 h	1 h	
	70% tB + 3% Quadrol	1 day	1 h	
Dehydration	tB PEG	2 days with daily change	10 min	
Clearing	BB-PEG	1 day	1 h	

### 2.7 Antibody information

**Table 2:** 

	Catalog number	Company	Dilution ratio	Host species
Primary antibody name				
Living Colors^®^ DsRed Polyclonal Antibody	632,496	Takara	1:200	Rabbit
Anti-Green Fluorescent Protein Antibody	GFP-1020	Aveslabs	1:200	Chicken
NeuN Rabbit mAb	A19086	ABclonal	1:200	Rabbit
Purified anti-β-Amyloid (6E10)	803,016	BioLegend	1:200	Mouse
Secondary antibody name				
Cy^™^3 AffiniPure^™^ Donkey Anti-Rabbit IgG (H + L)	711-165-152	Jackson ImmunoResearch	1:200	Donkey
Alexa Fluor^®^ 488 AffiniPure^™^ Donkey Anti-Chicken IgY (IgG) (H + L)	703-545-155	Jackson ImmunoResearch	1:200	Donkey
Cy^™^5 AffiniPure^™^ Donkey Anti-Mouse IgG (H + L)	715-175-151	Jackson ImmunoResearch	1:200	Donkey
Cy^™^5 AffiniPure^™^ Donkey Anti-Chicken IgY (IgG) (H + L)	703-175-155	Jackson ImmunoResearch	1:200	Donkey

#### 2.7.1 Antibodies used for DAT-Cre;Ai9 and VIP-Cre;Ai9 mouse brains (half or whole brain)

**Table 3:** 

Primary antibody	Dilution and incubation time	Secondary antibody	Dilution and incubation time
Living Colors^®^ DsRed Polyclonal Antibody	1:200 5 days	Cy^™^3 AffiniPure^™^ Donkey Anti-Rabbit IgG (H + L)	1:200 5 days

#### 2.7.2 Antibodies used for rabies viral tracer injected mouse brains (half or whole brain)

**Table 4:** 

Primary antibody	Dilution and incubation time	Secondary antibody	Dilution and incubation time
Living Colors^®^ DsRed Polyclonal Antibody	1:200 5 days	Cy^™^3 AffiniPure^™^ Donkey Anti-Rabbit IgG (H + L)	1:200 5 days
Anti-Green Fluorescent Protein Antibody	1:200 5 days	Alexa Fluor^®^ 488 AffiniPure^™^ Donkey Anti-Chicken IgY (IgG) (H + L)	1:200 5 days

#### 2.7.3 Antibodies used for YFV-mVenus viral tracer injected mouse brains (half or whole brain)

**Table 5:** 

Primary antibody	Dilution and incubation time	Secondary antibody	Dilution and incubation time
Anti-Green Fluorescent Protein Antibody	1:200 5 days	Cy^™^5 AffiniPure^™^ Donkey Anti-Chicken IgG (H + L)	1:200 5 days

#### 2.7.4 Antibodies used for Alzheimer’s disease model mouse brains (half or whole brain)

**Table 6:** 

Primary antibody	Dilution and incubation time	Secondary antibody	Dilution and incubation time
NeuN	1:200 5 days	Cy^™^3 AffiniPure^™^ Donkey Anti-Rabbit IgG (H + L)	1:200 5 days
Purified anti-β-Amyloid (6E10)	1:200 5 days	Cy™5 AffiniPure^™^ Donkey Anti-Mouse IgG (H + L)	1:200 5 days

### 2.8 iDISCO passive immersion procedure for 30 μm mouse brain sections

The protocol is an adaptation of the standard iDISCO method, with modifications primarily in the incubation durations. The procedure is succinctly outlined below.

#### 2.8.1 Non-methanol pretreatment

Fixed samples are washed in PTx.2 at room temperature for 5 min, twice. They are then incubated in 1xPBS with 0.2% Triton X-100 and 20% DMSO at 37°C for 2 h, followed by a second incubation in 1xPBS containing 0.1% Tween-20, 0.1% Triton X-100, 0.1% Deoxycholate, 0.1% NP40, and 20% DMSO at 37°C for 2 h. The procedure ends with another wash in PTx.2 at room temperature for 5 min, repeated twice.

#### 2.8.2 Staining

Samples are first incubated in Permeabilization Solution at 37°C for 1 h, followed by blocking in Blocking Solution at 37°C for 2 h. They are then incubated overnight with the primary antibody in PTwH with 5% DMSO and 3% Donkey Serum at 37°C. Afterward, the samples are washed in PTwH four to five times, each for 10 min. Subsequent incubation with the secondary antibody is done in PTwH with 3% Donkey Serum at 37°C, also overnight. The procedure concludes with a final wash in PTwH, four to five times, each for 10 min.

**Table 7:** 

Primary antibody	Dilution and incubation time	Secondary antibody	Dilution and incubation time
Chicken x GFP	1:500 overnight	Cy^™^3 AffiniPure^™^ Donkey Anti-Chicken IgG (H + L)	1:200 overnight

#### 2.8.3 Clearing

Samples were first incubated for 1 h in 10 mL of a 50% v/v tetrahydrofuran/water (THF) solution. Following this initial incubation, they were subjected to a 5-min incubation in 10 mL of 80% THF/water, and subsequently twice for 5 min each in 100% THF. The samples were then placed in dichloromethane (DCM) for 2 min. Finally, they were incubated in 18 mL of dibenzyl ether (DBE) until they became clear, approximately 1 h, and thereafter stored in DBE at room temperature.

### 2.9 Light-Sheet Microscopy and animation

iPEGASOS or PEGASOS cleared whole brain or half brain were imaged with the Cleared Tissue Light-Sheet (CTLS) microscope (3I Inc) (visible laser lines: 488,561,670 nm). The samples were immersed in BB-PEG clearing medium and scanned. A tiling Light-Sheet tiled at multiple positions within the field of view was used to illuminate the sample, and the sample was scanned with a 1.5 × /0.25NA objective axially at a 1 μm or 5 μm step size to image the sample in 3-D. All raw image data were collected in a lossless 16-bit TIFF files. 3D reconstruction images were generated using Slidebook (3I) and Imaris (Bitplane).

### 2.10 Viral injections

Mice were nasally anesthetized with continuous 1 ~ 2% isoflurane and placed in a rodent stereotaxic (Leica Angle Two for mouse) and secured at the head using ear bars. A three-axis micromanipulator guided by a digital atlas was used to locate to bregma and lambda and set bregma as origin for coordinates. The following injection coordinates for targeting secondary motor cortex (M2) (AP: −0.22; ML: −0.83; DV: −1.18) and retrosplenial cortex, granular area (RSCg) (AP: −2.46; ML: −0.2; DV: −0.93) were used. All values are given relative to the bregma. A small drill hole was made in the skull above the injection site, exposing the pia surface. Use fine forceps to carefully remove the pia above the injection site. Then lowered the glass pipette (tip diameter, ~20–30 μm) into brains. A Picospritzer (General Valve) was used to pulse virus from the glass pipette into the brain at a rate of 20–30 nL min^–1^ with 10-ms pulse durations. The Pipette tip remained in the brain for 5 min after injection to prevent virus backflow. Once the injection pipette was withdrawn, the mouse was removed from the stereotaxic machine, and the incision was closed with tissue adhesive (3 M Vetbond). Mice were returned to their home cages to recover.

To map input connections of M2-projecting RSCg neurons, 0.1 μL of retroAAV2-Cre virus (with a titer of 4.5 × 10^12^ genomic units per ml, Addgene) was delivered into the M2 region of wild-type C57BL/6 mice to target M2-projecting RSCg neurons. RetroAAV2-Cre can retrogradely transport into the RSCg and express Cre selectively in M2-projecting RSCg neurons. Then, 0.1 μL of AAV8-DIO-TC66T-2A-eGFP-2A-oG (0.1 μL, 9.5 × 10^12^ genome units per ml, SALK institute custom reagent) was delivered into the RSCg during the same surgery session. After 3 weeks, 0.2  μL of EnvA-SADΔG-DsRed rabies virus was injected into the same RSCg location. 9 days later, the mice were perfused.

For YFV-mVenus injected brains, the procedure was akin to that described above, with the sole distinction being the injection of 0.2 μL of YFV-mVenus (at a titer of 5.20 × 10^10^ genome units per ml, provided by UCI CNCM viral core) into both the dorsal subiculum and its overlaying cortex on the same day. Six days post-incubation, the mice were perfused, and their brains were extracted for either iPEGASOS or PEGASOS processing.

Mice of either sex were used for experiments and had free access to food and water in their home cages before and after surgeries. Genetically modified rabies viruses used for the experiments are deletion-mutant rabies viruses and are based on a vaccine strain (SADB19). The rabies viruses were made at the University of California, Irvine, with required cell lines and seeding viruses from E. Callaway’s group at the Salk Institute for Biological Studies.

### 2.11 Signal intensity measurement for different clearing method

#### 2.11.1 For iPEGASOS and PEGASOS comparison using YFV-mVenus injected samples

To evaluate the signal intensity between samples processed with iPEGASOS and PEGASOS, images (A1), (A2), (B1), and (B2) from Supplementary Figure S4. were analyzed. For sample N129, 100 fluorescently labeled cell somas in the Cy5 channel (A2) were identified manually using selection tool and designated as regions of interest (ROIs). The mean intensity for all individual ROIs was quantified using ImageJ. These identical ROIs were also utilized to assess the intensity in the green channel of sample N129 in (A1). In the case of sample N130, where signals were not visually discernible, 100 ROIs were randomly selected within the area depicted in (B1), and these same size ROIs were applied to analyze the region in (B2). The Mann–Whitney test was used to compare the summed intensity of Cy5 + mVenus in two samples, yielding a *p*-value <0.0001.

#### 2.11.2 For iPEGASOS and PEGASOS comparison using DAT-Cre;Ai9 samples

For this analysis, maximum intensity projections (MIPs) spanning the medial to lateral axis of whole hemispheres from DAT-Cre;Ai9 brains were analyzed for both iPEGASOS and PEGASOS processed samples. A predefined region of interest (ROI), represented in Supplementary Figure S2A and consistently sized across all samples, was designated for mean fluorescence intensity evaluation using ImageJ. This ROI was strategically positioned over the substantia nigra, which is readily identifiable due to its distinct, intensely bright appearance. A total of four samples (*N* = 4) were evaluated for each condition, and statistical significance was assessed using a Mann Whitney test with a *p*-value <0.05 considered indicative of a significant difference. All four samples were imaged with same setting.

#### 2.11.3 Comparison of iDISCO and iPEGASOS clearing methods in Tie2-GFP mouse brain sections

For these experiments, 30 μm coronal brain sections from the Tie2-GFP mouse line (endothelial cells are labeled with GFP in this strain) (Motoike et al., 2000) were selected for their suitability in quantitative analysis. The EGFP-expressing blood micro-vessel signal is uniformly distributed across the entire section, with no local absences or unevenness making it ideal for our analysis purpose. The iDISCO and iPEGASOS protocols were employed; their results were compared. We utilized a Chicken anti-GFP primary antibody paired with a Donkey anti-Chicken Cy3-labeled secondary antibody for immunostaining in both experimental groups, maintaining identical incubation periods across all staining steps to ensure comparability. The immunostaining signal was shifted to the Cy3 channel, enabling us to systematically examine the effects of both iDISCO and iPEGASOS on the preservation of endogenous fluorescence signals (GFP) as well as immunolabeled signals (Cy3). Upon completion of the staining and clearing processes, a uniform imaging setting was applied to capture all samples, preserving consistency in visualization. The ImageJ threshold function was then utilized to delineate and select stained blood vessels within the red channel, facilitating the measurement of mean gray intensity of the signals across the entire brain slice. These designated regions of interest (ROIs) were subsequently analyzed in the green channel to quantify the endogenous GFP signal’s intensity. For each experimental condition, we analyzed a total of six sections that span the anterior to posterior position of the brain. The anterior–posterior positions of these sections are closely matched between the two protocol conditions, ensuring comparability in the quantification of vessel intensity across the entire brain sections. Statistical analysis was conducted using a Wilcoxon matched-pairs signed rank test to determine significance between the two datasets, with a *p*-value <0.05 indicative of a significant difference.

In all quantitative figures, the Mean and Standard Deviation (SD) are displayed on the bar graphs to provide a clear representation of the data’s central tendency and variability.

#### 2.11.4 6E10 signal intensity and % area with signal quantification

For 6E10 signal intensity and density quantification, a single tiff image, comprising 200 planes merged through Maximum Intensity Projection, was utilized. Selected brain regions were cropped, and ImageJ’s “Measurement” and “Analyze Particles” functions were employed to quantify mean signal intensity and the percentage area with signal within these regions.

## 3 Results

### 3.1 Amplifying fluorescent signals in transgenic reporter mouse brains through iPEGASOS enhancement

**Figure 1:**
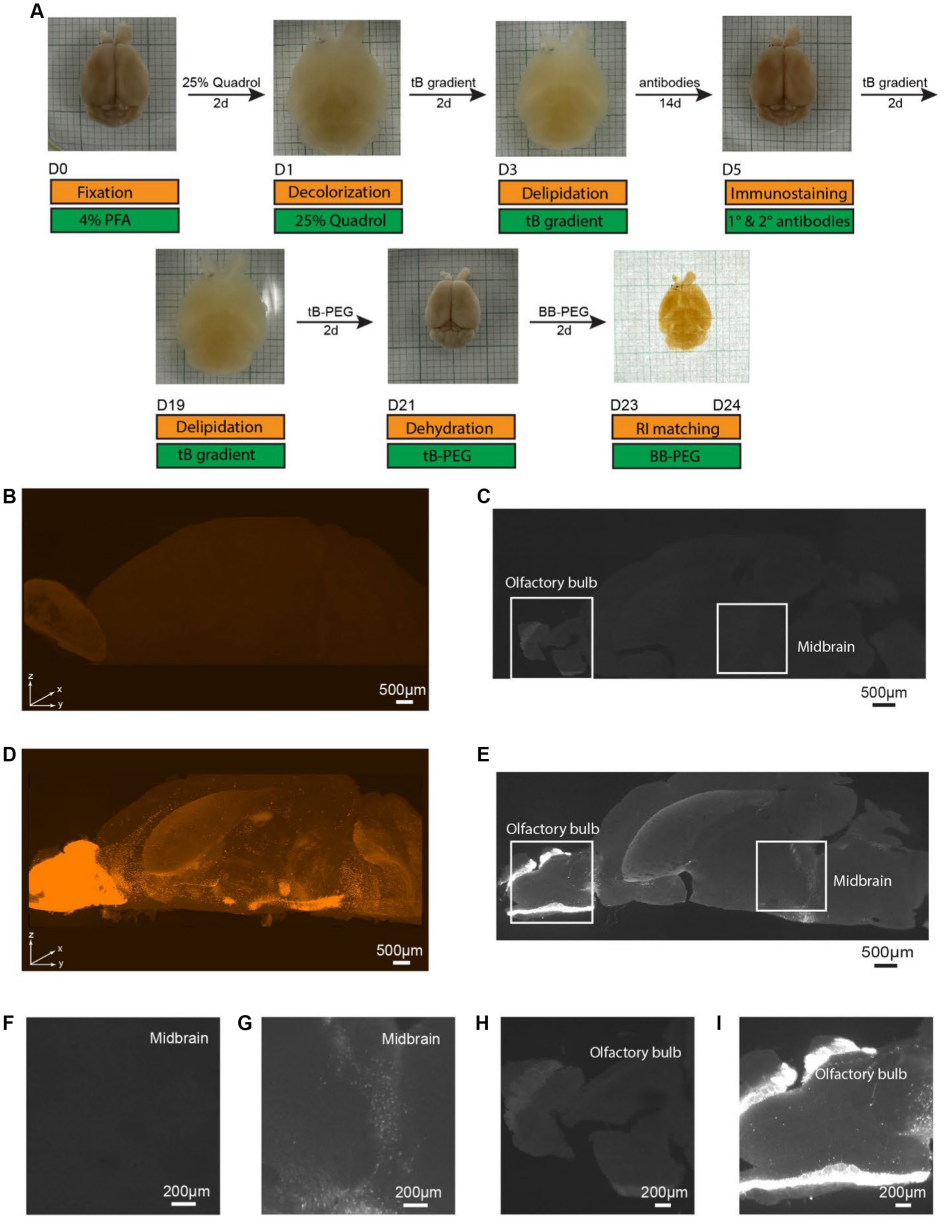
Specific single cell resolution immunostaining of whole brain cleared samples using iPEGASOS, visualized by Light-Sheet imaging of DAT-Cre;Ai9 mouse hemisphere. **(A)** iPEGASOS process. Top row: the state of the whole mouse brain at each stage of iPEGASOS. Bottom: in gold and green boxes, we depict each step, solution, and duration for the iPEGASOS processed whole mouse brain. **(B)** The autofluorescence background depicted in the top-volume view of the brain that is cleared but without immunostaining. The sample was imaged using 1.5x,0.37NA objective, Light-Sheet Microscopy, with the dorsal side facing up. **(C)** Lack of signal in a brain not incubated with primary and secondary antibodies shown in a horizontal optical plane in the midbrain region, showing very low background autofluorescence. **(D)** Antibody staining signal in a top volume view of a whole brain cleared following immunostaining using rabbit x DsRed antibody. Strong signal is seen in the midbrain region and olfactory bulb, indicating that the process allows immunolabeling of deep brain regions. **(E)** Specific DAT promoter driven tdTomato signal amplified with anti-DsRed antibody seen in a horizontal optical plane in midbrain region. **(F,G)** Higher magnified views of the white boxes at the midbrain region in **(C,E)** show low background for the no antibody control **(F)** and single-cell resolution signal for the antibody-treated brain **(G)**, respectively. **(H)** Higher magnified views of a single optical plane at olfactory bulbs in panel **C**, showing low autofluorescence background. **(I)** Higher magnified views of a single optical plane at olfactory bulbs in panel **E**.

To ascertain the necessity of immunostaining for signal visualization, we performed a comparative study using PEGASOS (clearing) and iPEGASOS (immunostaining + clearing) on transgenic mouse line DAT-Cre;Ai9 (Zhuang et al., 2005). These mice are characterized by tdTomato expression in dopaminergic neurons, mainly in the midbrain areas and olfactory bulb (Cave and Baker, 2009). We selected the DAT-Cre;Ai9 mouse model due to its limited number of DAT-positive cells within specific brain regions. This unique feature enables us to circumvent potential antibody insufficiency issues and leverage the model to showcase the specificity of antibody staining. Mouse brains of the same age and sex were cleared with iPEGASOS (Figure 1A) or PEGASOS (Supplementary Figure S1A) and scanned using same settings. Robust signals are observed in the 3D reconstructed hemispheres, achieved through immunostaining with a rabbit anti-DsRed antibody (which presents the staining signal in the Cy3 channel) using iPEGASOS (Figure 1D). Notably, this technique precisely captures single-cell resolution signals in the deep structure midbrain (MB) (Figures 1E,G) and olfactory bulb (Figure 1I) in the optical sections. Conversely, in brains subjected to clearing without immunostaining (PEGASOS), only a faint autofluorescence background is detected in the reconstructed 3D brain (Figure 1B) and in individual optical sections of the midbrain (Figures 1C,F) and olfactory bulb (Figure 1H). Quantitative analysis of the selected regions revealed significantly higher fluorescence signals in the iPEGASOS-processed DAT-Cre;Ai9 brain tissue compared to those processed with PEGASOS (Supplementary Figure S2). This outcome conclusively affirms the ongoing necessity of immunostaining, even when the sample already contains genetically expressed fluorescent signals.

**Figure 2:**
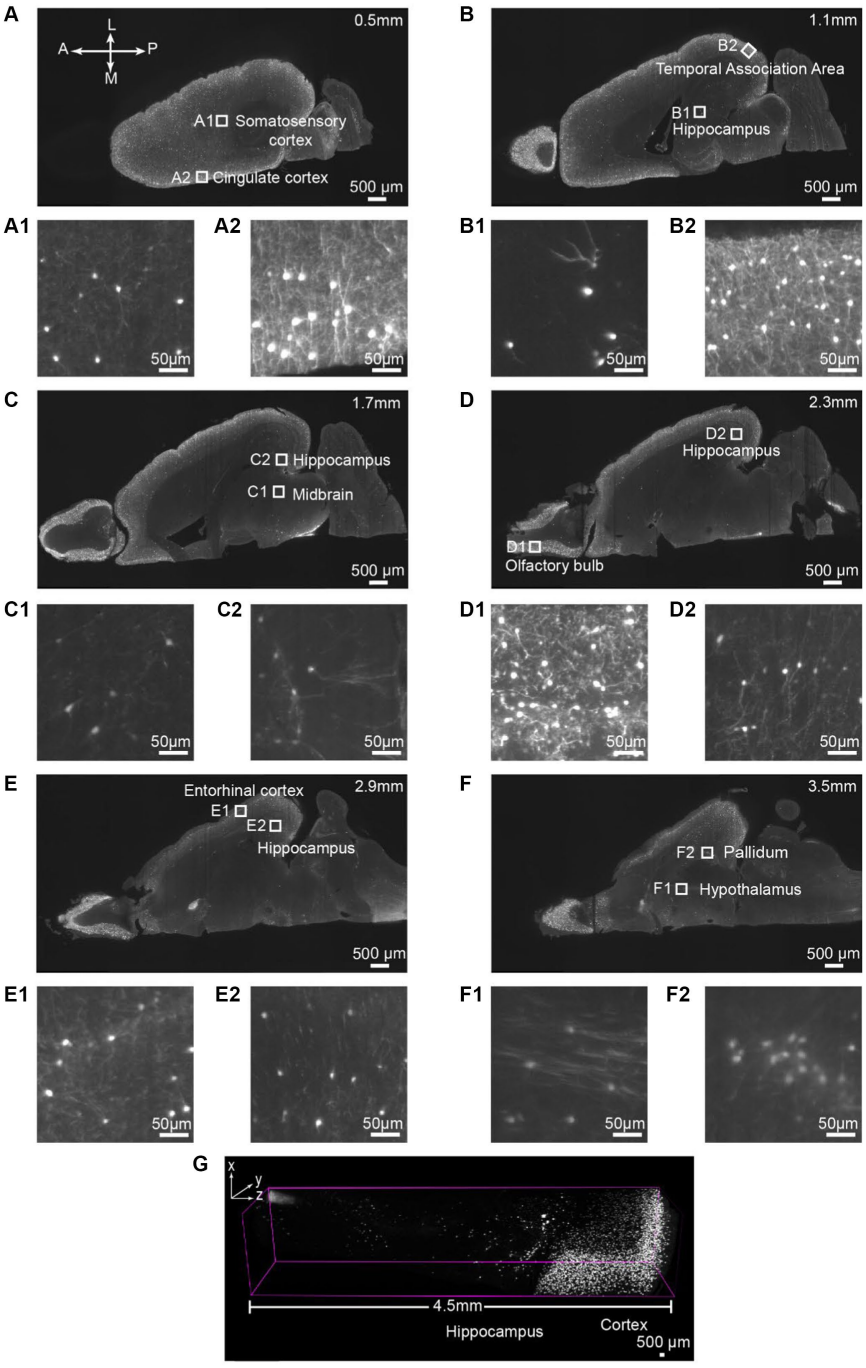
Single-cell resolution imaging can be achieved throughout the entire brain from the dorsal (Top imaging plane) to the ventral (bottom imaging plane) plane of the VIP-Cre;Ai9 mouse hemisphere. **(A–F)** Various depths of horizontal optical planes from a VIP-Cre; Ai9 mouse hemisphere cleared and immunostained using iPEGASOS and rabbit x DsRed antibody. It was acquired from a 1.5x Light-Sheet Microscopy. A to F are optical planes: 0.5, 1.1, 1.7, 2.3, 2.9 and 3.5 mm away from the dorsal part (top) of the brain. A, Anterior; P, Posterior; M, Medial; L, Lateral. **(A1,A2–F1,F2)** Single-cell resolution zoom-in of white-boxed regions in **(A–F)**. **(G)** A digitally segmented cross-section of the brain features a 4.5 mm-tall column, organized from the dorsal (right) to ventral regions (left), including the cortical areas, hippocampus, thalamus, and midbrain.

Next, we aim to assess the antibody binding capability of iPEGASOS against antigens distributed ubiquitously throughout the entirety of the brain, spanning from the cortical surface to the deep brain regions. We stained and cleared the hemisphere from a vasoactive intestinal polypeptide (VIP)-cre;Ai9 mouse using DsRed antibody (present the staining signal in Cy3 channel) and iPEGASOS. Serial optical slices were captured using a 1.5x, 0.37NA objective Light-Sheet Microscopy along the dorsal-ventral axis of the brain. Abundant and intense signals were observed in the cerebrum (including the neocortex, hippocampus and olfactory bulb), with a lesser presence in the brainstem and cerebellum regions (Figures 2A–G). The white boxed regions in Figures 2A–F are enlarged to show the high resolution of single-neuron-level images (Figures 2A1,A2–F1,F2). The signal is sharp at the dorsal side (Figures 2A1,A2) but decreases in quality while approaching the most ventral level of the brain (Figures 2F1,F2,G). This is due to the imaging configuration rather than antibody tissue penetration: the brain is imaged in a single pass horizontally with the dorsal side facing emission objective lens – without flipping the brain over for another imaging pass, followed by digitally stitching together the two imaging sets. Despite this, single-cell resolution is accomplished even at most ventral part of the brain where optical aberration is most significant. Furthermore, we compared signal quality by positioning either the dorsal or ventral sides of the VIP-Cre; Ai9 mouse brain facing upward, using identical imaging settings. The comparison focuses on the 166th planes below the top, revealing consistent fluorescence intensity and single-cell resolution between the two conditions (Supplementary Figure S3).

**Figure 3:**
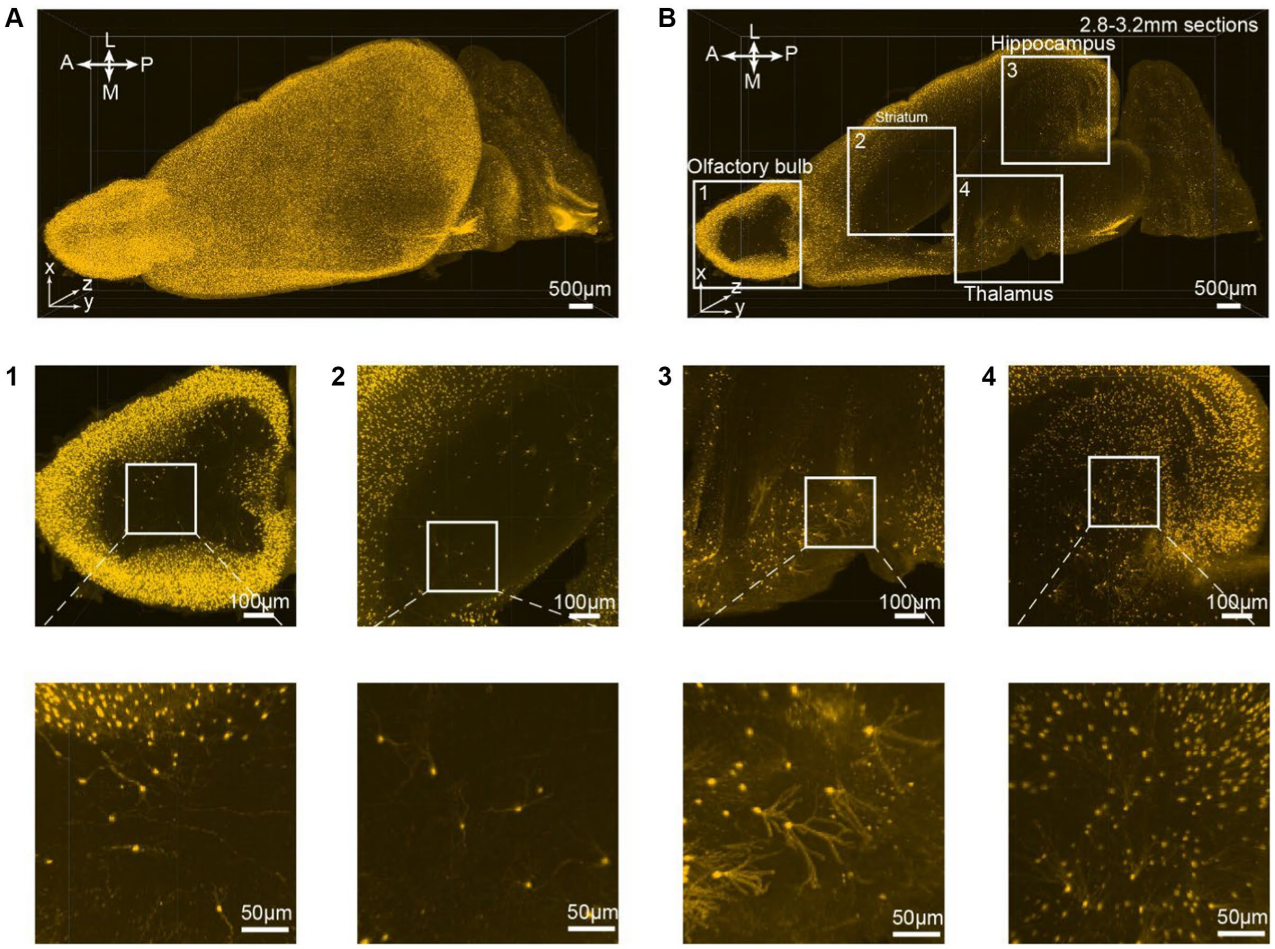
3D rendering of the VIP-Cre;Ai9 mouse hemisphere showing dendritic-level resolution. **(A)** A rendered hemisphere viewed from the dorsal top. A, Anterior; P, Posterior; M, Medial; L, Lateral. **(B)** Maximum intensity projection created from 133 optical planes (400 μm thickness). **(B1–B4)** Top: Boxed regions in B are further enlarged to generate **(B1–B4)**. Bottom: These images are the higher magnifications of the boxed region indicated on top.

All consecutive optical slices from the VIP-Cre;Ai9 mouse hemisphere are digitally reconstructed into 3D images (Figure 3A; Supplementary Movies S2). Subsequently, 133 optical planes were extracted, resulting in a 0.4 mm thickness brain section (Figure 3B; Supplementary Movies S1). Higher magnification views are depicted at different brain regions ranging from the periphery to the inner part of the brain, including the olfactory bulb (Figure 3B1), striatum (Figure 3B2), thalamus (Figure 3B3), and hippocampus (Figure 3B4). The iPEGASOS-processed VIP-Cre;Ai9 hemisphere unveils robust fluorescent signals originating from individual VIP neuronal cell bodies and their associated processes, including dendrites. The opposite hemisphere from the same VIP-Cre;Ai9 mouse was cleared using the PEGASOS method for comparative purposes. These control preparations only yield weak signals (Supplementary Figures S1B–G). Hence, iPEGASOS proves its applicability to stain mouse brains by delivering accurate labeling that extends to dendritic-level resolution without introducing artifactual or mislabeled signals.

### 3.2 Achieving comprehensive 3D brain-wide neural circuit mapping in intact mouse brain samples via iPEGASOS

The diverse functions of brains, ranging from the simplest reflexes to complex cognitive and emotional processing, are embedded in neural circuits. To delineate these circuits and unveil their roles for brain functions, viral tracers are injected intracranially in specific regions guided by stereotaxis to map local and distal connections. Whole brain mapping of circuits using Light-Sheet Microscopy has more stringent requirements than mapping the relative locations of cell bodies because the size of the dendritic and axonal neuronal processes is much smaller than that of cell somas. iPEGASOS gives a superior signal-to-noise ratio for viral tracers injected brains to the extent that we can image both neurons and processes clearly. To demonstrate the utility of iPEGASOS for large-scale neural circuit mapping, we stained and cleared brains injected with Adeno-associated virus (AAV) helper and pseudo-typed rabies virus (Sun et al., 2014; Callaway and Luo, 2015; Lin et al., 2021; Ye et al., 2022).

**Figure 4:**
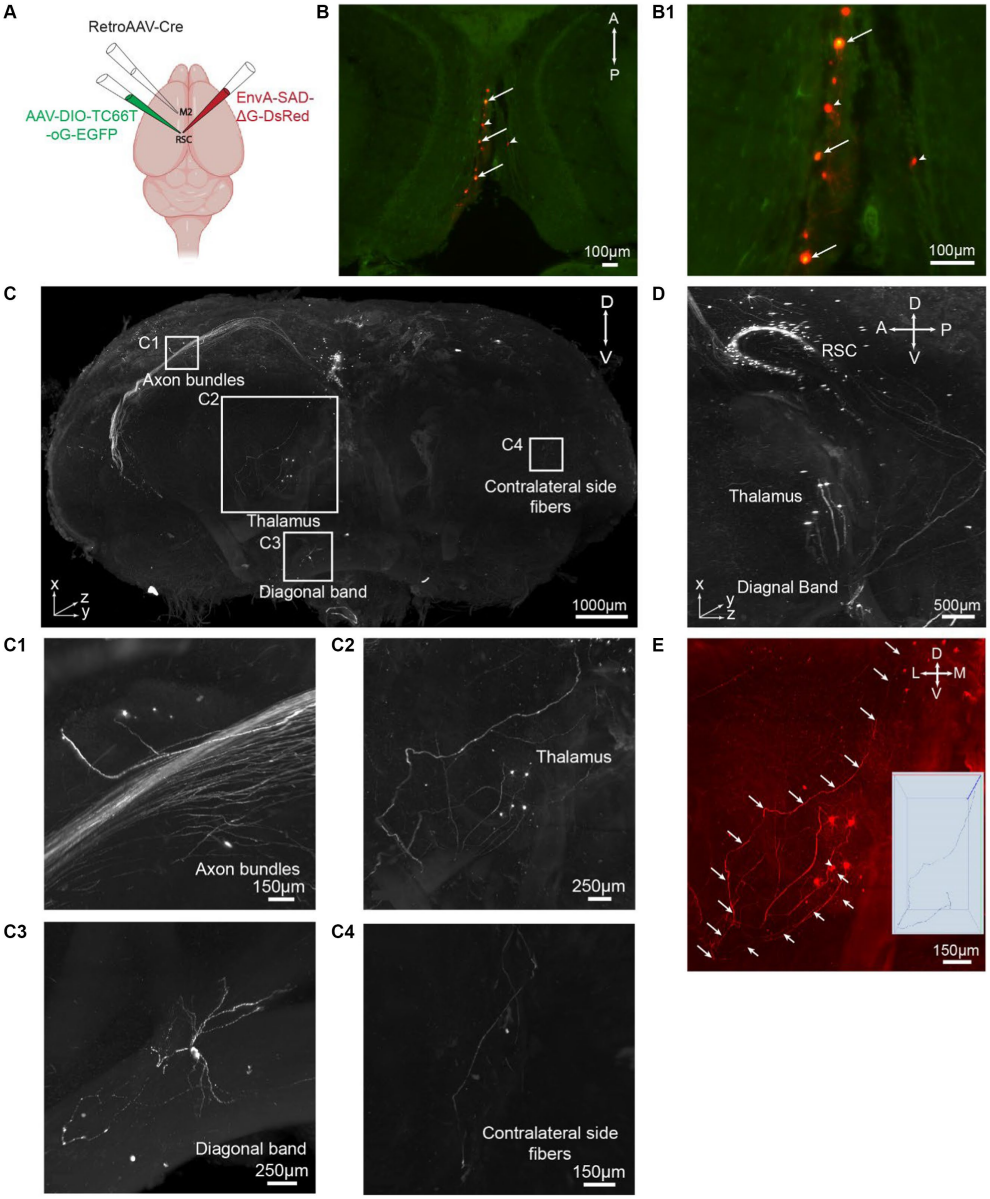
iPEGASOS allows pseudorabies virus-based whole-brain neural circuit mapping. **(A)** The schematic drawing for the viral injection approach. **(B)** Starter cells are pointed with arrows in a single optical section (horizontal view). Cells with arrowheads are input neurons in RSCg. **(B1)** At a higher magnification, we zoom in on the starter cells, revealing the colocalization of DsRed from the rabies viral tracer and EGFP from AAV. **(C)** A 3D rendering provides a front view of a brain injected with a rabies viral tracer. The signal (DsRed) highlights input cells connecting to M2-projecting RSCg cells. The brain was imaged coronally. TH, Thalamus; DB, Diagonal band; RSC, Retrosplenial cortex. A, anterior; P, posterior; M, medial; L, lateral; D, dorsal; V, ventral. **(C1–C4)** Zooming in on the white boxed regions in panel **C**, we observe axon bundles **(C1)**, single neurons and axons from thalamus input cells **(C2)**, single-cell soma and neural processes from diagonal band input cells **(C3)**, and axons projecting to the contralateral side of the injection site **(C4)**. **(D)** A side view of the 3D brain provides a close-up, highlighting the regions of the RSC, TH, DB. **(E)** A single axon is traced from their origin in the thalamic soma to their endpoints in the RSC.

To demonstrate the applicability of iPEGASOS for pathway-specific retrograde tracing, we utilized this technique to stain and clear brains injected with AAVs and rabies viral tracers (Figure 4A). We first injected retrogradely transporting RetroAAV-Cre (rAAV2-retro-Cre) (Tervo et al., 2016) into the secondary motor cortex (M2) through intracranial stereotaxic injection. Cre recombinases were expressed in M2-projecting retrosplenial cortex (retrosplenial cortex, granular area = RSCg) neurons. Subsequently, Cre-dependent AAV helper (AAV-DIO-TC66T-oG-EGFP) was injected into RSCg, inducing the expression of TC66T (Avian receptor for EnvA pseudotyped rabies virus entry), oG [optimized native rabies glycoprotein essential for glycoprotein (G)-deleted rabies virus entry], and EGFP in M2-projecting RSCg neurons. After 3 weeks of AAVs incubation, EnvA-pseudotyped glycoprotein-deleted rabies virus (EnvA-RVΔG-DsRed) were injected into RSCg, allowing them to enter AAV helper-infected cells. The RVΔG from the starter neurons (both EGFP and DsRed expression) can retrogradely spread to directly connected presynaptic cells, expressing DsRed. As oG is absent in these presynaptic cells, the RVΔG cannot spread further across synapses. This monosynaptic RV tracing system enables comprehensive mapping of neural circuit inputs to RSCg neurons projecting to the secondary motor cortex (M2). Nine days after rabies injection, we perfused the mice and processed these brains with iPEGASOS, and imaged them with a 1.5x, 0.37NA Light-Sheet Microscope. Starter cells in the RSCg deep layer are identified by their co-localization of DsRed and EGFP signals (Figures 4B,B1). The monosynaptic input cells for M2-projecting RSCg neurons are displayed in the red channel. Neural somas of these input cells are prominently visible across the entire brain, with a substantial concentration in the RSCg itself, thalamus, medial septum diagonal band, and various cortical regions, including the visual, auditory, and somatosensory cortex (Figures 4C,C1–C4; Supplementary Figure S5). Thick axon bundles are fluorescently labeled (Figure 4C). Predominantly, these axon bundles belong to intrinsic input cells originating from the retrosplenial cortex (RSC), while a subset of axons originates from input cells within cortical regions (Figure 4C1). Additionally, individual axons are evident between the RSC and other input regions, like the thalamus and diagonal band (DB) (Figures 4C2,C3). These observations indicate the exceptional transparency achieved throughout the entire brain, accompanied by remarkable lateral (1 μm/pixel) and axial (1 μm/pixel) resolution (Figures 4C,D). To determine whether we can trace individual axons, we focused on the anterior thalamic region because its connection with RSCg is relatively sparse and is thus easier to trace along single axons. With a 1.5 x objective and 4.33 digital zoom-in, we were able to trace a single axon from a single soma in the thalamus that projects to RSC for around 4 mm (Figure 4E).

To demonstrate the superior signal intensity offered by iPEGASOS over PEGASOS in the context of viral-assisted reporter gene expression, we conducted a quantitative analysis of the fluorescent signal intensities stem from YFV-mVenus (Li et al., 2021), an anterograde viral tracer, in mouse brains processed with either of the two clearing methods. This comparison is detailed in Supplementary Figure S4, where the enhanced efficacy of iPEGASOS in intensifying fluorescent signals is evident. Following intracranial injection of YFV-mVenus into the dorsal subiculum and overlaying cortical areas (Visual cortex), the expected mVenus fluorescence was assessed both at the injection site and within monosynaptically connected regions (YFV-mVenus was only incubated for 6 days). The brain processed with iPEGASOS was stained with a chicken anti-GFP antibody and shifted mVenus detection to the Cy5 channel (Supplementary Figure S4A). By selecting a region adjacent to the injection site, we quantified the intensities of both the inherent mVenus and the antibody-amplified Cy5 signals (Supplementary Figures S4A1,A2). The findings revealed that iPEGASOS substantially augmented the fluorescence signal intensity of the virally expressed protein (Supplementary Figures S4C,D), underscoring its efficacy in enhancing visualization of viral tracer expression, compared to PEGASOS.

In summary, the brains processed using the iPEGASOS technique achieves a notable degree of clearing, with an intensified signal that is helpful for detecting individual cell bodies and tracing single axons. Coupled with volumetric imaging using a Light-Sheet Microscope with adequate lateral and axial resolution, iPEGASOS enables us to undertake tasks such as cell detection, axon tracing, and neural circuit mapping.

### 3.3 Tracking the progression of brain beta-amyloid accumulation in 5xFAD mice (6 to 17 months) using iPEGASOS

The iDISCO method has previously been utilized to stain amyloid-beta in the 2xTG (APPswe, PSEN1dE9) Alzheimer’s disease mouse model, demonstrating its utility in visualizing amyloid pathology (Liebmann et al., 2016). Building upon this foundation, we sought to explore the potential of the iPEGASOS method in capturing the age-dependent progression of beta-amyloid accumulation in a more aggressive Alzheimer’s disease (AD) model. Our study focuses on the 5xFAD mouse model, known for its rapid development of pronounced amyloid pathology, making it an ideal candidate for such advanced imaging techniques. By conducting immunostaining for beta-amyloid (Aβ) in 5xFAD mice across a range of ages, from 6 to 17 months, and using control wild-type (WT) mouse brains for comparison (one sample for each age), we aim to demonstrate the feasibility of iPEGASOS for tracking the progression of temporal and spatial dynamics of Aβ accumulation.

In the 5xFAD mouse model, transgenes for human β-Amyloid precursor protein (APP) and PSEN1 (a core protein in the γ-secretase complex) are expressed, encompassing a total of five mutations associated with Alzheimer’s disease: Swedish (K670N/M671L), Florida (I716V), and London (V717I) mutations in APP, along with M146L and L286V mutations in PSEN1 (Oakley et al., 2006). This model rapidly develops pronounced amyloid pathology, characterized by elevated intraneuronal Aβ42 levels starting at approximately one and half months of age. Extracellular amyloid deposition begins around 2 months, with plaques distributed throughout the hippocampus and cortex by 6 months. In older mice, plaques extend to the thalamus, brainstem, and olfactory bulb but are notably absent from the cerebellum (Oakley et al., 2006; Forner et al., 2021). The monoclonal antibody 6E10, one of the earliest commercially available anti-amyloid monoclonal antibodies against Aβ, is widely used in Alzheimer’s disease research. The epitopes for 6E10 correspond to residues 4–10 of Aβ, allowing it to recognize specific linear segments of Aβ 1–42. Unlike some antibodies that target spatial conformations or higher-order structures of a peptide, 6E10 can detect various forms of Aβ, including full-length peptides and smaller fragments. This characteristic makes it valuable for detecting amyloid plaques in histological studies and quantifying Aβ levels in biochemical assays (Baghallab et al., 2018; Readel et al., 2023).

**Figure 5:**
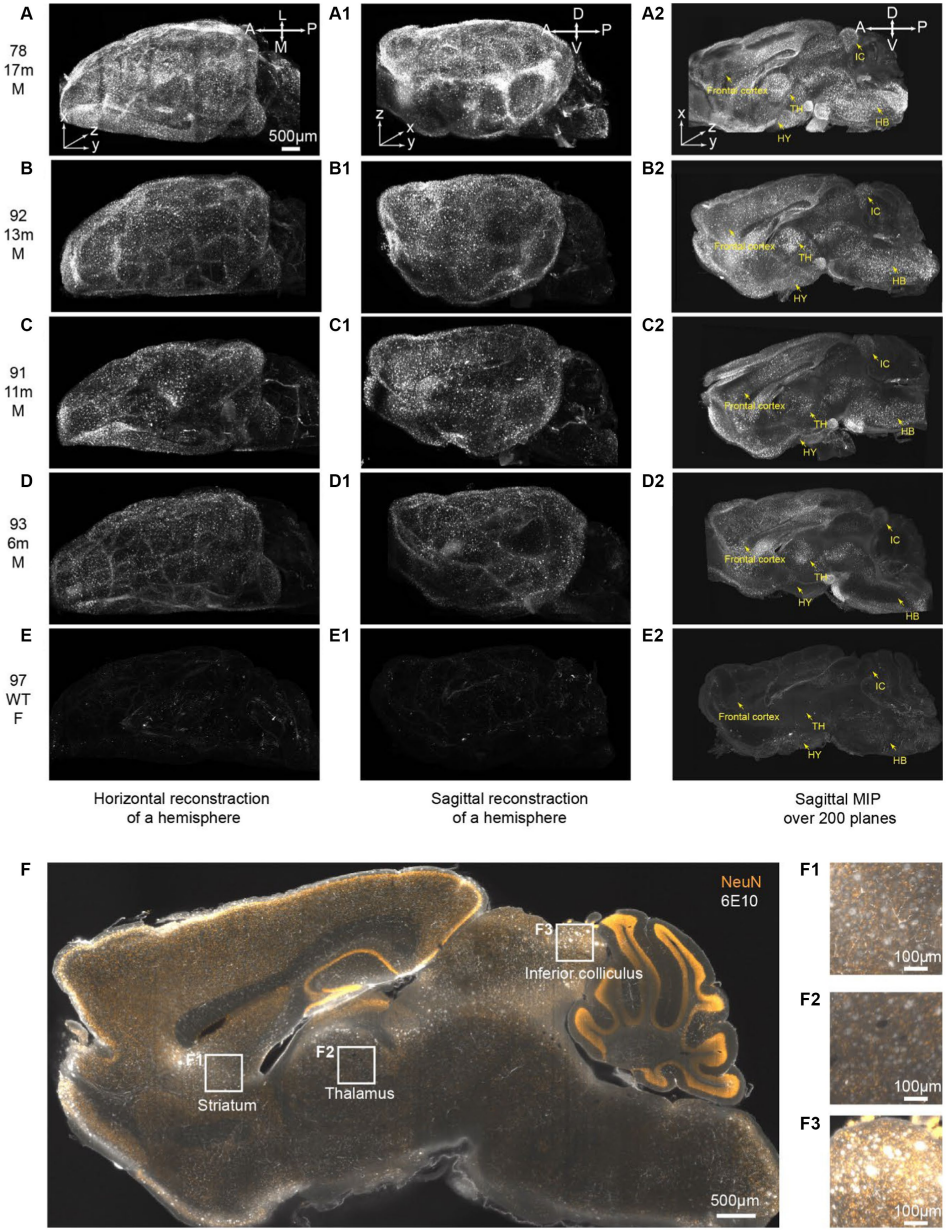
iPEGASOS captures the progression of beta-Amyloid accumulation in 5xFAD ranging from 6 m to 17 m of age. **(A–E)** Top views of 3D reconstructed brains are presented for different conditions: **(A)** 5xFAD, 17-month-old, male; **(B)** 5xFAD, 13-month-old, male; **(C)** 5xFAD, 11-month-old, male; **(D)** 5xFAD, 6-month-old, male; and **(E)** Wildtype, 8-month-old, female. The white signal corresponds to 6E10 antibody staining for beta-amyloid peptide. **(A–E)**, **(A1–E1)**, and **(A2–E2)** shared the same scale bar. The brains were imaged sagittally. A, anterior; P, posterior; M, medial; L, lateral; D, dorsal; V, ventral. **(A1–E1)** Side view of the corresponding brains in **(A–E)**. **(A2–E2)** A maximum intensity projection is generated from 200 optical planes spanning 1.2 mm. Yellow arrows highlight key structures: frontal cortex, hypothalamus (HY), thalamus (TH), inferior colliculus (IC), and hindbrain (HB).**(F)** A single optical section is displayed from the brain of a 13-month-old male 5xFAD mouse. The yellow signal represents NeuN staining, while the white signal corresponds to 6E10 staining. **(F1–F3)** Higher magnifications at **(F1)** striatum, **(F2)** thalamus, and **(F3)** inferior colliculus.

In this study, the entire brains were cut medially, and subsequent iPEGASOS procedures were carried out on male 5xFAD model mouse hemispheres spanning a range of ages from 6 to 17 months. This assessment involved utilizing 6E10 antibodies to quantify Aβ deposition. We also include an 8-month-old female C57BL/6 WT mouse brain that underwent the same processing procedure as a negative control. All brains were imaged sagittally using 1.5x Light-Sheet Microscopy with consistent exposure time and laser power settings for gain adjustments. To provide a comprehensive depiction of the entire brain, we presented both the dorsal (Figures 5A–E) and lateral views (Figures 5A1–E1) of the 3D brain (xy: 2um/pixel; z:6um/pixel). Control WT mouse brain exhibits minimal background signals with partial staining of blood vessels observed (Figures 5E,E1,E2). In contrast, for 5xFAD mice, Aβ plaques encompass the entire cortex at 6 months of age (Figures 5A–D,A1–D1). The spatial progression of Aβ plaques is revealed when looking at maximum intensity projection (MIP) from 200 sagittal planes (total thickness: 1.2 mm) starting from the medial midline (Figures 5A2–E2). Gold arrows point to the frontal cortex, hypothalamus (HY), thalamus (TH), inferior colliculus (IC), and hindbrain (HB) (Figures 5A2–E2), showing age-related accumulation of plaques. We quantified signal mean intensity within specific brain regions (frontal cortex, thalamus, hypothalamus, inferior colliculus, and hindbrain), normalizing to WT mice. An age-dependent increase in 6E10 signal intensity was observed in 5xFAD mice (Supplementary Figure S7). Additionally, we assessed the percentage of area with 6E10 signal within the circumscribed brain regions, noting a higher proportion with aging in 5xFAD mice (Supplementary Figure S7). The same iPEGASOS procedure was applied to other types of AD mouse models, specifically 3xTG and APP-KI mice. The staining and clearing of these models resulted in similar quality, as shown in Supplementary Figure S6.

The 13-months-old 5xFAD brain (brain ID: #92) underwent co-staining with the NeuN antibody, a recognized neuronal marker. A single optical plane of this staining is displayed in Figure 5F, with subsequent zoomed-in views highlighting regions such as the striatum, thalamus, and inferior colliculus. Our observations reveal that a significant portion of beta-amyloid peptides occupies the extracellular space, giving rise to plaques ranging from 20 μm to 50 μm. Notably, certain plaques exhibit colocalization with NeuN staining (Figures 5F1–F3). It is important to acknowledge that due to the widespread and abundant presence of the NeuN antigen throughout the entire brain, achieving uniform whole-brain NeuN immunostaining may present a significant challenge. The penetration depth of the antibody into the brain tissue is hindered as it depletes during its journey from the periphery towards the core of the mouse brain. Given this constraint and considering our specific antibody dilution ratio, we can only be confident that iPEGASOS is effective for staining NeuN in single mouse hemispheres, but extending this assurance to whole-brain staining requires further validation.

## 4 Discussion

Visualizing molecules and cells within the entire 3D structure of the brain is an important advance in neurobiology by providing a global view of brain circuitry and constituent cell types. Tissue clearing is a recent technical innovation that confers transparency to otherwise opaque tissues by reducing the RI difference between different components of tissues. The power of this approach is leveraged by volumetric imaging using Light-Sheet Microscopy to fully capture intricate neural structures in intact whole brain or thick brain samples. Our iPEGASOS protocol effectively preserves the integrity of molecular-genetically targeted fluorescent signals while facilitating the subsequent clearing of tissue for optical imaging. We have leveraged technical advancement from the whole-mount immunolabeling protocol iDISCO and combined them with the PEGASOS tissue clearing protocol to devise iPEGASOS: this enables uniform and complete antibody diffusion throughout all tissue depths for whole mouse brain and confers fluorescence protection along with superior transparency.

iDISCO is particularly effective for whole-mount immunolabeling. However, its tissue-clearing phase (based on the earlier 3DISCO method) comes at the cost of considerably attenuating of fluorescent signal intensity by over 90% (Nudell et al., 2022). The developers of that method contend that endogenous fluorophore protection is dispensable, as signal visualization can be achieved by immunolabeling. However, preserving the endogenous fluorescent signal is an important goal for tissue having genetically targeted fluorescent labeling. For iDISCO, the reduction in the fluorescent signal primarily occurs during the methanol dehydration step before immunostaining (quench genetically targeted signal) and the refractive index matching step using dibenzyl ether after the immunostaining process (quench genetically targeted signal & immunostaining signal) (Nudell et al., 2022). While the decline in genetically targeted fluorescence during the dehydration phase may not be a significant concern, the loss of immunolabeled signals during the refractive index matching step is certainly undesirable. PEGASOS [the polyethylene glycol (PEG)-associated solvent system] has desirable features for tissue clearing based on three major criteria: transparency, fluorescence preservation, and tissue applicability. As an organic-solvent-based clearing method, PEGASOS renders tissue transparency in nearly all types of tissue, including the spleen, liver, heart, and even bone and teeth, that are hard to clear by other clearing methods. By comparison, soft tissues that lack high levels of endogenous pigments, like a mouse brain, can be cleared with relative ease. In contrast to the majority of organic solvent-based clearing protocols that tend to diminish fluorescence, the PEG component in PEGASOS serves as a protective shield for fluorophore proteins by preventing denaturation. Furthermore, it effectively scavenges free radical groups within the solvent, which otherwise quench signals. This unique feature leads to improved signal intensity following a one-week incubation in the final PEG-containing refractive index matching solution (Jing et al., 2018). Both GFP and tdTomato fluorescence have been shown to retain approximately 70% of their original intensity after completing all PEGASOS clearing steps (Jing et al., 2018). This leads to the hypothesis that combining the iDISCO staining protocol with the PEGASOS clearing protocol (iPEGASOS) could yield samples with higher intensity than those processed solely with iDISCO.

In our study, we conducted a comparative analysis to investigate the retention of fluorescent signals in Tie2-GFP (Motoike et al., 2000) mouse brain sections (endothelial cells are labeled with GFP in this strain) following the processing with either iDISCO or iPEGASOS, as depicted in Supplementary Figures S8A,B. Both sets of brain sections were subjected to immunostaining under identical conditions, using the same antibody dilution and incubation duration. The key methodological difference between the two groups was the differing protocols applied during the clearing phase. The results revealed a contrast in the preservation of endogenous GFP signals between the two methods. In the iDISCO processed samples, the endogenous GFP signal was nearly entirely quenched, as evidenced in Supplementary Figures S8A2,D. In comparison, sections processed with iPEGASOS retained visible GFP signals, indicating a lesser degree of quenching (Supplementary Figures S8B2,D). Moreover, when examining the immunostained signals displayed in the red channel, iPEGASOS-treated samples exhibited a higher intensity compared to those processed with iDISCO (Supplementary Figures S8A1,B1,D). These findings support the advantages of the iPEGASOS method for not only preserving but also enhancing the fluorescent signals from both endogenous expression and immunostaining in brain tissue sections. Furthermore, based on our own comparative observations from PEGASOS and iPEGASOS cleared brains, particularly in VIP-Cre;Ai9 and DAT-Cre;Ai9 mice, revealed that iPEGASOS cleared brains exhibited higher tdTomato fluorescence signal intensity under identical imaging settings. Based on these observations, we conclude that iPEGASOS-cleared brains may exhibit more than 70% of the original intensity. Consequently, by combining iDISCO’s immunostaining with PEGASOS clearing, we leverage the deep tissue labeling capacity of iDISCO and the superior fluorescence preservation of PEGASOS. This combined approach is to address the observed limitations in fluorescence retention with iDISCO alone, as well as enhancing the overall quality and utility of whole-brain imaging by PEGASOS.

It is worth mentioning that in our study, we observed that the endogenous fluorescent signal (signals expressed from transgenic mouse line or viral-assisted reporter gene) is nearly undetectable in certain mouse brains, as illustrated in Figure 1B and Supplementary Figures 2B, 4A1,B1. This observation necessitates a consideration of the intrinsic expression features of the transgenic mouse lines or viral tracers employed in our experiments. It is possible that specific lines or tracers inherently produce weaker fluorescence signals, which may contribute to the apparent diminution of signal intensity following the clearing process. The variability in fluorescent protein expression levels, rather than any intrinsic limitation of the PEGASOS protocol, could explain the observed signal attenuation. Moreover, the reduced visibility of signals does not impugn the effectiveness of PEGASOS; signal quenching is a widespread phenomenon among solvent-based clearing techniques. Notably, our results demonstrate that PEGASOS surpasses iDISCO in terms of signal preservation, as evidenced by Supplementary Figures S8A2,B2. These findings highlight the critical role of iPEGASOS, particularly when endogenous fluorescent signals are obscured following PEGASOS processing, reinforcing the necessity for iPEGASOS in such scenarios.

The sequence of immunolabeling and tissue clearing is crucial for ensuring effective antibody penetration and achieving optimal transparency in tissue samples. In the context of 3D visualization, particularly for complex structures like the mouse brain, this sequence becomes even more critical due to the brain’s size and lipid content. A recent attempt to visualize apical periodontitis within the mouse jawbone in 3D employed an alternative immunostaining protocol combined with PEGASOS clearing (Tazawa and Sasaki, 2023). This approach involved whole-mount immunolabeling inserted between the decolorization and delipidation steps of the PEGASOS process. While this method proved simple and effective for the mouse jawbone, a structure significantly smaller and less lipid-dense than the brain, it is less suitable for brain tissue. In the brain, the absence of a delipidation step before staining would likely restrict antibody diffusion, given the brain’s higher lipid content. To address these challenges, our staining protocol draws from the iDISCO method, specifically designed for whole-mount staining of mouse brains. The iPEGASOS approach we employed maintains a critical sequence: first, it removes RI-mismatched components like pigments and lipids, then proceeds to immunostaining, and concludes with RI matching. This initial removal of lipids and pigments significantly increases the brain’s permeability, facilitating deeper antibody penetration into the tissue.

To address concerns regarding the potential impact of imaging configuration on signal intensity in the ventral area of the brain, we reimaged the samples with either the dorsal or ventral side facing up, employing identical imaging settings. Subsequent analysis, focusing on the 166th planes below the brain surface, revealed that fluorescence intensity and signal clarity did not exhibit significant changes between the two conditions. For future investigations involving time-course changes or whole-brain-wide regional comparisons of signal intensity (e.g., Aβ deposition), it might be beneficial to consider alternative imaging strategies. One such strategy involves stitching together the top half of the sample from two imaging sessions with flipped configurations. This approach could offer a comprehensive solution to mitigate potential biases introduced by the imaging perspective.

While we acknowledge recent advancements in the whole-mount immunostaining field, we want to highlight the distinction and potential advantages of our method, iPEGASOS. Before delving into specific details of comparing different protocols, it is essential to provide a brief introduction to the strategies that have been employed in the field (Yau et al., 2023): 1) Increasing antibody (Ab) availability: This involves elevating antibody concentrations or reducing antibody depletion by temporarily inhibiting antibody–antigen binding (Murray et al., 2015; Susaki et al., 2020; Lai et al., 2022). 2) Enhancing tissue diffusivity: This strategy focuses on increasing effective diffusivity in the tissue through elevating tissue permeability (Renier et al., 2014; Zhao et al., 2020) or reducing the distance for diffusion (Cai et al., 2019; Ku et al., 2020). 3) Advective transport of antibodies: this innovative approach involves increasing antibody transport through advective methods, such as electrophoresis (Kim et al., 2015). Both iDISCO and iPEGASOS staining procedures fall into strategy 2, prioritizing enhanced sample permeabilization to improve antibody penetration into the deep regions of the brain. Compared with the CUBIC-HistoVIsion (CUBIC-HV) method that mainly utilizes strategy 1, iPEGASOS has some technical advantages. The iDISCO/iPEGASOS staining method, modeled after traditional histology techniques, uses common and inexpensive reagents for sample processing, eliminating the need for specialized lab equipment. Notably, it remains compatible with the standard two-step indirect fluorescent immunostaining process (primary antibody and secondary antibody staining). This compatibility ensures that the quality of immunolabeling observed on tissue sections can serve as a reliable predictor for the corresponding quality in whole-mount immunolabeling using the iDISCO-based method, highlighting the practical significance of this technical advantage (Renier et al., 2014). However, the CUBIC-HV protocol does not encourage staining of 3D samples using the standard two-step process but uses direct fluorescent immunostaining using the antibodies that are directly conjugated with Alexa Fluor® dyes or FabuLightTM Fab fragment secondary antibodies, which can be costly in terms of antibody supplies. Furthermore, the CUBIC-HV method has a particular limitation regarding its incompatibility with Alexa Fluor^®^ dye AF488 (Susaki et al., 2020). In addition, although immunostaining time is comparable between CUBIC-HV and iPEGASOS, our iPEGASOS/PEGASOS approach demonstrates superior transparency in clearing nearly all types of tissues, including bone and teeth, which are hard to clear with other methods, including CUBIC-HV (Jing et al., 2018). The inclusion of the PEG component in iPEGASOS protects immunostaining fluorescent signals despite being an organic solvent-based clearing method. In conclusion, iPEGASOS has advantages in antibody compatibility, cost-effectiveness, and superior tissue-clearing capability when compared with other methods, including CUBIC-HV. These technical strengths allow iPEGASOS to be considered as a valuable choice for whole-mount immunostaining and clearing applications by researchers.

In terms of 2D sectioning-based 3D reconstruction techniques, recent advancements, including serial two-photon tomography (STPT/TissueCyte) (Ragan et al., 2012), fluorescence micro-optical sectioning tomography (fMOST) (Gong et al., 2013), and block-face serial microscopy tomography (FAST) (Seiriki et al., 2017) represent innovative solutions that integrate imaging and automatic sectioning processes. These block-face serial imaging methods, equipped with internally installed microtomes or microslicers, address the challenges associated with labor-intensive manual sectioning and potential image alignment and registration errors. In the case of TissueCyte, its workflow involves post-fixing brain samples in 4% PFA and embedding them in 3 to 5% agarose (~ one day). Additional steps may be incorporated into specific protocols to enhance stiffness, such as soaking the agarose-embedded brain in acrylamide or sodium borohydrate (an additional 2 days) (Ragan et al., 2012). No need for decolorization, delipidation, and dehydration steps in block-face serial imaging methods is advantageous as it mitigates tissue shrinkage issues commonly encountered in many organic solvent-based clearing methods. This advantage contributes to the preservation of the original tissue structure, addressing a concern frequently observed in other clearing approaches. PEGASOS, the antecedent to iPEGASOS, is reported to exhibit a tissue shrinkage issue (~40% for the brain). However, it is noteworthy that despite shrinkage, it manages to avoid detectable distortion of the internal brain structure as mentioned earlier (Jing et al., 2018). While the absence of tissue clearing steps in block-face serial imaging methods may render them incompatible with thick-tissue immunostaining, even though these methods permit staining with dyes. Thus, the lack of immunostaining capability may restrict their application for signal intensification or staining of molecular markers unavailable through transgenic or viral delivery approaches. Taken together, immunostaining of the whole brain tissue, constituting the most time-consuming step in the workflow, remains valuable. In addition, the refractive index matching (clearing) step, a key component of many tissue clearing methods, typically requires an additional 2 days and is often reversible. This flexibility enables a unique hybrid approach: clearing the brain, imaging it using a light-sheet microscope, and subsequently reverting it to its opaque, non-cleared state for imaging using block-face serial imaging methods. Additionally, one advantage of tissue clearing with light-sheet imaging is that it allows multiple imaging sessions of the same sample, offering flexibility in capturing low-resolution images for a quick signal check of the whole brain and subsequently focusing on specific regions for higher-resolution scanning. Following clearing, samples can remain in the clearing medium for at least a year as mentioned earlier, showcasing the stability and longevity of the technique; we have samples cleared for more than 2 years without significant signal loss. Another limitation of block-face serial imaging techniques is their long imaging time. In comparison to the relatively short imaging times of cleared tissues with light-sheet microscopy (approximately two to three hours), TissueCyte may take ~7 days, and fMOST may require ~19 days for whole-brain imaging. Notably, FAST stands out as a more time-efficient option, capable of imaging the entire brain within a range of 2.4 to 10 h per brain per color channel, depending on objective settings. While FAST offers rapid whole-brain imaging, it is worth noting that its self-assembly requires expertise, including the adjustment of optical axes and the alignment of confocal and stage movement planes. Operators must possess sufficient knowledge to ensure safe and proper operation of lasers and to prevent injuries and accidents (Seiriki et al., 2019). It is important to acknowledge that TissueCyte, fMOST, and FAST microscopes are not as widely available in the imaging centers of academic institutions, compared with Light-Sheet Microscopes.

In conclusion, iPEGASOS provides a strategic integration of whole-mount immunostaining with PEGASOS, yielding intact, transparent mouse brains that exhibit superior fluorescent signals relative to PEGASOS-cleared brains. The immunostaining steps confer significant signal amplification and visualization. This achievement not only enables the retention of signal quality but also allows for the exploration of non-transgenic neurochemical markers or proteins. Our work exemplifies the tangible advantages of employing iPEGASOS with Light-Sheet-based volumetric imaging. This combined approach enables comprehensive profiling of the distribution of genetically defined cell types and facilitates the mapping of neural circuits across entire individual mouse brains with unprecedented precision thus leading to an enhanced understanding of the mouse brain. Lastly, our study also demonstrates the capabilities of the technique in capturing the progression of pathology associated with Alzheimer’s disease.

## Data availability statement

The original contributions presented in the study are included in the article/Supplementary materials, further inquiries can be directed to the corresponding author.

## Ethics statement

The animal study was approved by the Institutional Animal Care and Use Committee and the Institutional Biosafety Committee of the University of California, Irvine. The study was conducted in accordance with the local legislation and institutional requirements.

## Author contributions

PG: Writing – original draft, Writing – review & editing. MR: Methodology, Software, Writing – review & editing. XL: Data curation, Writing – review & editing. TH: Writing – review & editing. HZ: Conceptualization, Supervision, Writing – review & editing. XX: Conceptualization, Funding acquisition, Supervision, Writing – review & editing.
