# Supplementary material for: Immunolabeling-compatible PEGASOS tissue clearing for high-resolution whole mouse brain imaging
Source: Front Neural Circuits. 2024 Apr 17;18:1345692. doi: 10.3389/fncir.2024.1345692 (PMC11061518; doi:10.3389/fncir.2024.1345692)
Supplement: Supplementary file 1 [file Data_Sheet_1.docx]

**A detailed step by step iPEGASOS protocol**

- The procedure involved transcardial perfusion with 4% paraformaldehyde (PFA) followed by extraction of mouse brains. Subsequently, the brains were immersed in 4% PFA overnight to 24 hours. The next step involved transferring the brains to 1x phosphate-buffered saline (PBS) for 1hr twice in preparation for further processing.
- If the brains had been stored in sucrose previously, they were required to undergo a series of transfers to fresh 1xPBS. Specifically, change to fresh 1xPBS ~5 to10 times within 48 hours before initiating the iPEGASOS protocol.
- For decolorization, delipidation, dehydration, and clearing incubation time, the form below served as a reference, drawing from the PEGASOS 2018 paper (1. Jing D, Zhang S, Luo W, Gao X, Men Y, Ma C, et al. Tissue clearing of both hard and soft tissue organs with the PEGASOS method. Cell Res. 2018 Aug;28(8):803–18.).


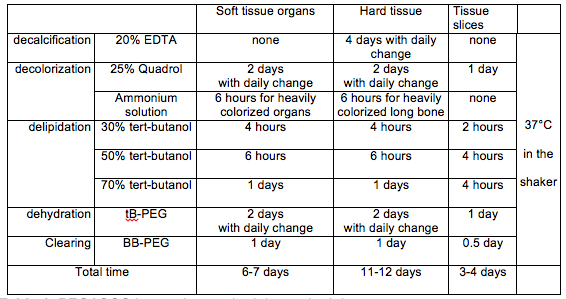


**STEP I: Day 1～2: Decolorization with Quadrol**

Reagents:

- 25% Quadrol (N,N,N',N'-Tetrakis(2-hydroxypropyl)ethylenediaminel) (dilute Quadrol with ddH2O, both volume ratio and weight ratio are ok.)

Methods:

- Place samples into 25%Quadrol for 1 or 2 days in 37-degree shaker depending on remaining blood of specific samples, change medium every day till medium won’t become yellow anymore.

**STEP II: delipidation (1^st^ round):**

- 30%, 50%, 70% Tert-Butanol (dilute with ddH2O, volume ratio) with 3% quadrol.
- Gradient Dilapidation with 30%, 50%, 70%Tert-Butanol at 37-degree shaker for 2 days (30% for ~4 hours, 50% for ~6 hours; 70% the rest of time).
- Tip: It is recommended to maintain a pH of ≥9.5 when making 30/50/70% Tert-Butanol. In cases where the pH does not reach the optimized value, Quadrol may be slightly increased, typically by adding 3-5% Quadrol.

**STEP III: iDISCO pretreatment:**

- Samples washed in PBS for 1 hr twice, and in PBS / 0.2% Triton X-100 (PTX.2) for 1 hr twice before further staining procedures.
- Pretreated samples incubated in PBS/0.2% Triton X-100 / 20% DMSO/ 0.3 M glycine (Permeabilization Solution) at 37 C for ~2days, then blocked in PBS/0.2% Triton X-100 / 10% DMSO / 3% Donkey Serum (blocking solution) at 37 C for 2 days.

**STEP IV: iDISCO staining:**

- Samples were washed in PBS/0.2% Tween-20 with 10 ug/ml heparin (PTwH), (When make 1L PTwH, add 1ml of 10mg/ml heparin stock solution.) for 1 hr twice, then incubated in primary antibody dilution in PTwH / 5% DMSO / 1% Donkey Serum at 37 C for 5 days. (syringe-filter the solution at 0.22μm.)
- Samples were then washed in PTwH (5 times a day) for 2 days, then incubated in secondary antibody dilutions in PTwH / 1% Donkey Serum at 37 C for 5days. Samples were finally washed in PTwH (5 times a day) for 2 days before clearing and imaging.

******Centrifuging secondary antibody solution at 20000g for 10 minutes can prevent formation of precipitates in the sample. Alternatively, you can syringe-filter the solution at 0.22μm.

**STEP V:** **Delipidation (2^nd^ round)**

- 30%,50%, 70% Tert-Butanol (dilute with ddH2O, volume ratio), add 3~5% quadrol.
- Gradient Dilapidation with 50%, 70%Tert-Butanol at 37-degree shaker for 2 days (30% 4 hours, 50% 6 hours; 70% 1 day).

**STEP VI: Dehydration**

- TB-PEG-MEM (70%Tert-Butanol + 25~27%PEG-MEM, volume ratio), add 3~5% quadrol.
- Final dehydration with TB-PEG-MEM at 37-degree shaker for 2 days, change medium each day (whole brain or half brains for 2 days; hippocampus only overnight).

**STEP VII: Clearing**

- BB-PEG-MEM (75%Benzyl benzoate + 20~22% PEG-MEM, volume ratio), add 3~5% quadrol.
- Tip: Dry the sample from TB-PEG and change a new tube before putting into BB-PEG.
- Immerse samples into the final clearing medium BB+PEG-MEM for at least 2 days (Note that please make fresh 200 ml of BB+PEG-MEM, shield from light but expose it to air ahead of time for 2 days (Tip: you could put it in a beaker and expose to the air in the first two days and then seal it.)
