# Supplementary material for: Immunolabeling-compatible PEGASOS tissue clearing for high-resolution whole mouse brain imaging
Source: Front Neural Circuits. 2024 Apr 17;18:1345692. doi: 10.3389/fncir.2024.1345692 (PMC11061518; doi:10.3389/fncir.2024.1345692)
Supplement: Supplementary file 2 [file Data_Sheet_2.pdf]

Supplementary Material

Immunolabeling-compatible PEGASOS Tissue Clearing for High-resolution Whole Mouse Brain Imaging

Pan Gao[1], Matthew Xavier Rivera[1], Xiaoxiao Lin[1], Todd C Holmes[2,4], Hu Zhao[3], Xiangmin Xu[1,4*]

[1]Department of Anatomy and Neurobiology, School of Medicine, University of California, Irvine, Irvine, CA, United States, [2]Department of Physiology and Biophysics, School of Medicine, University of California, Irvine, Irvine, CA, United States, [3]Chinese Institute for Brain Research, Beijing, China, [4]Center for Neural Circuit Mapping, University of California, Irvine, Irvine, CA, United States

*** Correspondence:**

Corresponding Author: Xiangmin Xu
xiangmix@hs.uci.edu

1 Supplementary Movies Legend

Movie S1. A VIP-Cre;Ai9 hemisphere stained and cleared with iPEGASOS. A maximum intensity projection (MIP) of several optical planes is zoomed in on various regions to observe cell morphology.

Movie S2. 3D rendering of entire hemisphere of the VIP-Cre; Ai9 mouse brain, stained and cleared with iPEGASOS. A flythrough of all optical planes of VIP-Cre;Ai9 hemisphere.

2 Supplementary Figure Legends

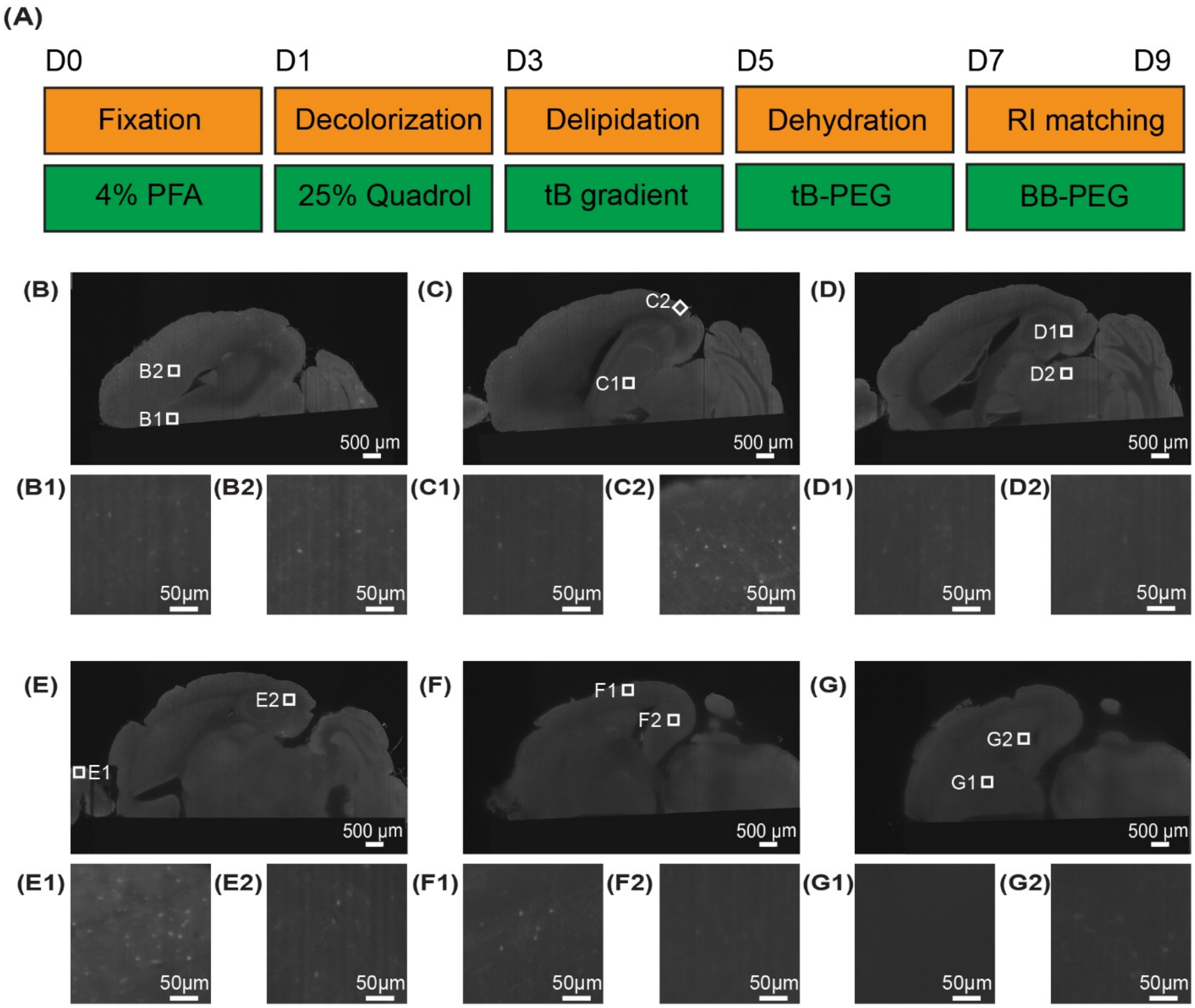
(A)
D0
D1
D3
D5
D7
D9
Fixation
Decolorization
Delipidation
Dehydration
RI matching
4% PFA
25% Quadrol
tB gradient
tB-PEG
BB-PEG
(B)
B2
B1
500 µm
(C)
C2
C1
500 µm
(D)
D1
D2
500 µm
(B1)
(B2)
(C1)
(C2)
(D1)
(D2)
50µm
(E)
E2
E1
500 µm
(F)
F1
F2
500 µm
(G)
G2
G1
500 µm
(E1)
(E2)
(F1)
(F2)
(G1)
(G2)
50µm

Supplementary Figure 1. PEGASOS-cleared brains only show weak fluorescent signals throughout the brain. (A) In gold and green boxes, we depict each step, solution, and duration for PEGASOS processed whole mouse brain. **(B-G)** Various depths of horizontal optical planes from a VIP-cre x Ai9 mouse hemisphere that were cleared using PEGASOS. It was acquired from a 1.5x Light-Sheet Microscopy. B to G are single optical planes: 0.5, 1.1, 1.7, 2.3, 2.9 and 3.5mm away from the dorsal part (top) of the brain. **(B1,2-G1,2)** Single-cell resolution zoom-in of white-boxed regions in B-G.

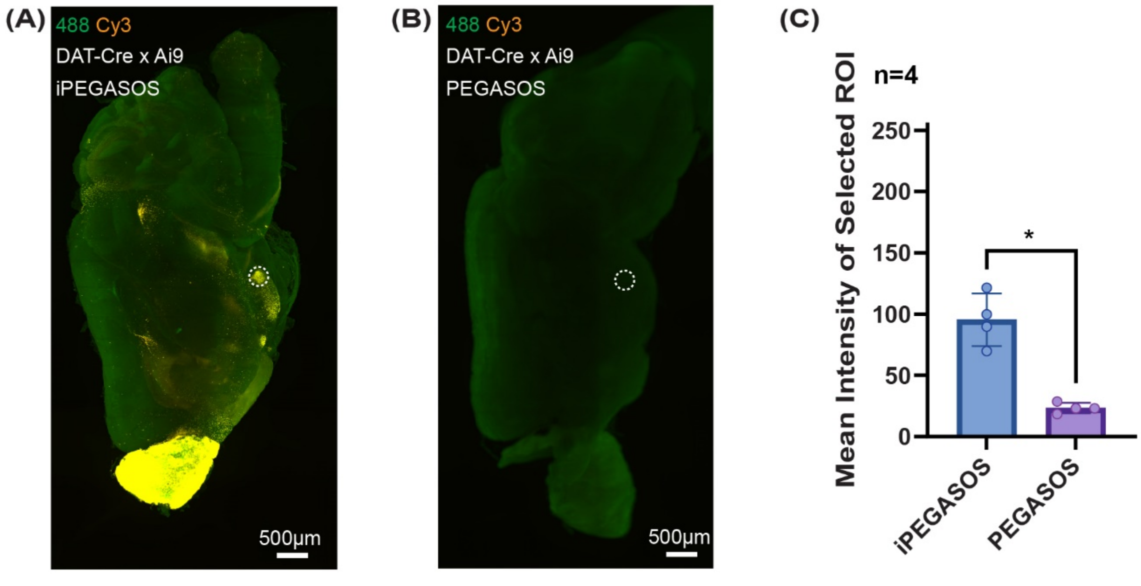
(A)
488 Cy3
DAT-Cre x Ai9
iPEGASOS
500μm
(B)
488 Cy3
DAT-Cre x Ai9
PEGASOS
500μm
(C)
n=4
Mean Intensity of Selected ROI
250
200
150
100
50
0
*
iPEGASOS
PEGASOS

Supplementary Figure 2. Fluorescence Signal Comparison in DAT-Cre;Ai9 Brains Cleared by iPEGASOS and PEGASOS. (A) Maximum intensity projection (MIP) of a hemisphere from a DAT-Cre;Ai9 brain stained with an anti-DsRed antibody and cleared using iPEGASOS. The dotted region indicates the area selected for mean fluorescence intensity measurement via ImageJ. (B) MIP of a hemisphere from a DAT-Cre;Ai9 brain cleared using PEGASOS, with the dotted region indicating the area used for mean intensity measurement. (C) Quantitative analysis of mean intensities demonstrates that iPEGASOS-cleared samples exhibit significantly higher fluorescence signals compared to those cleared with PEGASOS in DAT-Cre;Ai9 hemispheres. Mann Whitney test with a p-value = 0.0286.

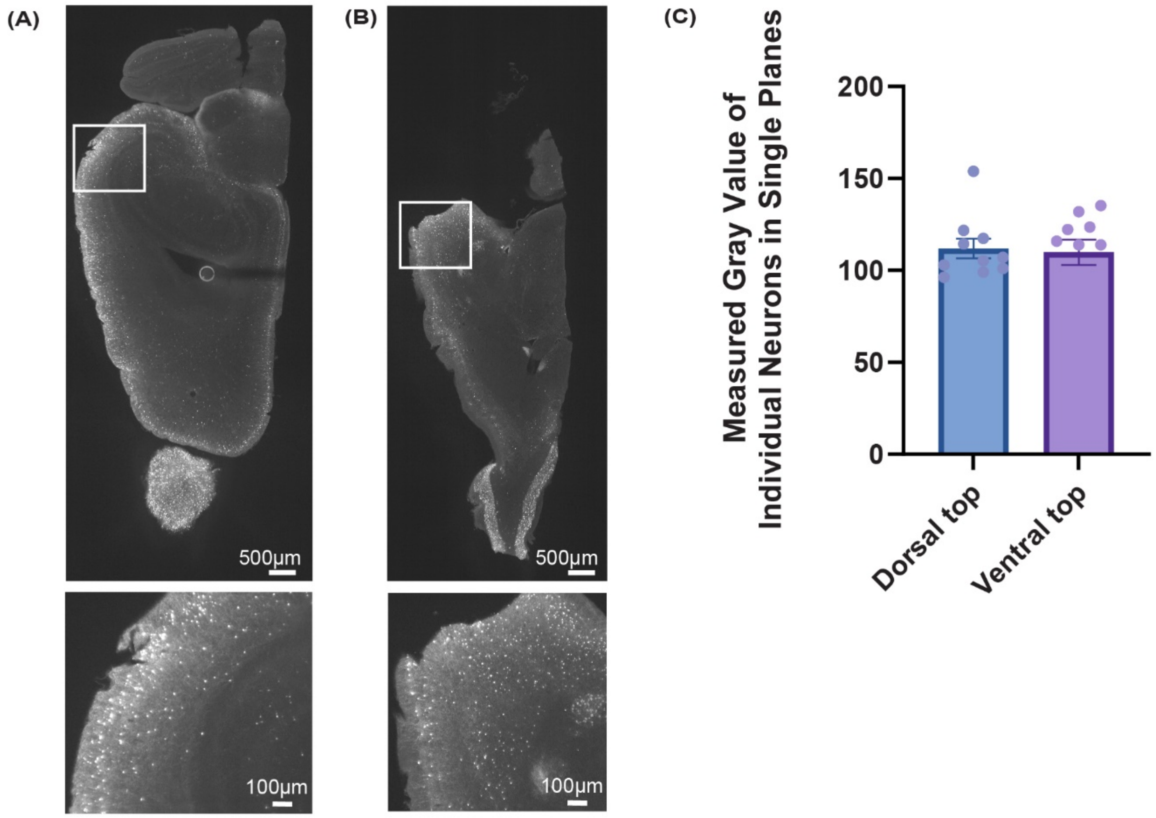
(A)
500µm
100µm
(B)
500µm
100µm
(C)
Measured Gray Value of Individual Neurons in Single Planes
200
150
100
50
0
Dorsal top
Ventral top

Supplementary Figure 3: Dorsal-Ventral Imaging Comparison for VIP-Cre;Ai9 mouse brain.

(A) Dorsal Imaging: The VIP-Cre;Ai9 mouse brain was imaged with the dorsal side facing up. The top panel displays the 166th single plane from the top, while the bottom panel provides a zoomed-in view for detailed examination. **(B)** Ventral Imaging: Imaging of the same brain with the ventral side facing up. The top panel shows the 166th single plane from the top, and the bottom panel offers a close-up perspective. **(C)** Quantitative analysis of individual neurons' intensity in both dorsal and ventral imaging conditions in (A) and (B), with measurements taken from 10 neurons for each condition.

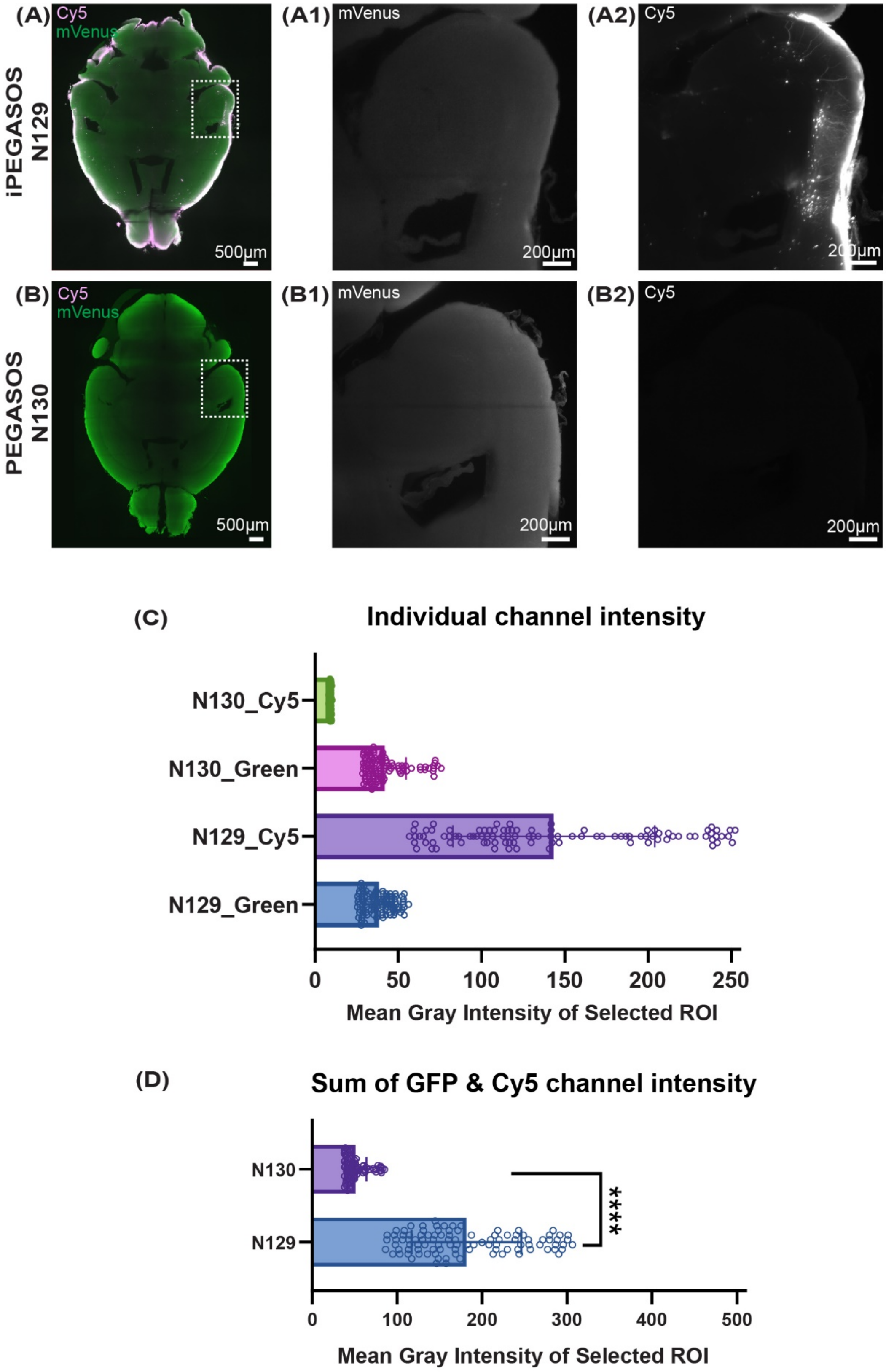

(A)
Cy5
mVenus
iPEGASOS
N129
500µm
(A1)
mVenus
200µm
(A2)
Cy5
200µm
(B)
Cy5
mVenus
PEGASOS
N130
500µm
(B1)
mVenus
200µm
(B2)
Cy5
200µm
(C)
Individual channel intensity
N130_Cy5
N130_Green
N129_Cy5
N129_Green
0
50
100
150
200
250
Mean Gray Intensity of Selected ROI
(D)
Sum of GFP & Cy5 channel intensity
N130
N129
****
0
100
200
300
400
500
Mean Gray Intensity of Selected ROI

Supplementary Figure 4: Comparative Visualization of YFV-mVenus in Mouse Brains Cleared by iPEGASOS and PEGASOS. (A) Mouse brain N129, injected with YFV-mVenus targeting the dorsal subiculum and overlying cortex, was processed with iPEGASOS and stained mVenus expression with an anti-GFP antibody in the Cy5 channel. The resulting image is a maximum intensity projection (MIP) comprising 10 optical sections. An enlarged view of the mVenus signal within the white-boxed region is presented in **(A1),** while **(A2)** focuses on the intensified Cy5 channel in the same region. **(B)** In parallel, mouse brain N130 received a similar YFV-mVenus injection and was cleared with PEGASOS, with the image displayed as an MIP of 10 optical sections corresponding closely to those of (A). The mVenus signal in the white-boxed area is shown in **(B1),** and the Cy5 channel signal in the same area is depicted in **(B2). (C)** To assess the efficacy of the staining processes, images (A1), (A2), (B1), and (B2) were analyzed for signal intensity differences. Specifically, in the Cy5 channel of sample N129, 100 fluorescently labeled cell somas were selected for intensity measurement to draw comparisons between iPEGASOS and PEGASOS processed samples. (D) The analysis revealed that the iPEGASOS processed sample N129 exhibited significantly higher overall intensity in both the mVenus and Cy5 channels compared to the PEGASOS processed sample, underscoring the enhanced signal intensification afforded by the iPEGASOS method. The Mann-Whitney test was used to compare the summed intensity of Cy5 + mVenus in two samples, yielding a p-value < 0.0001.

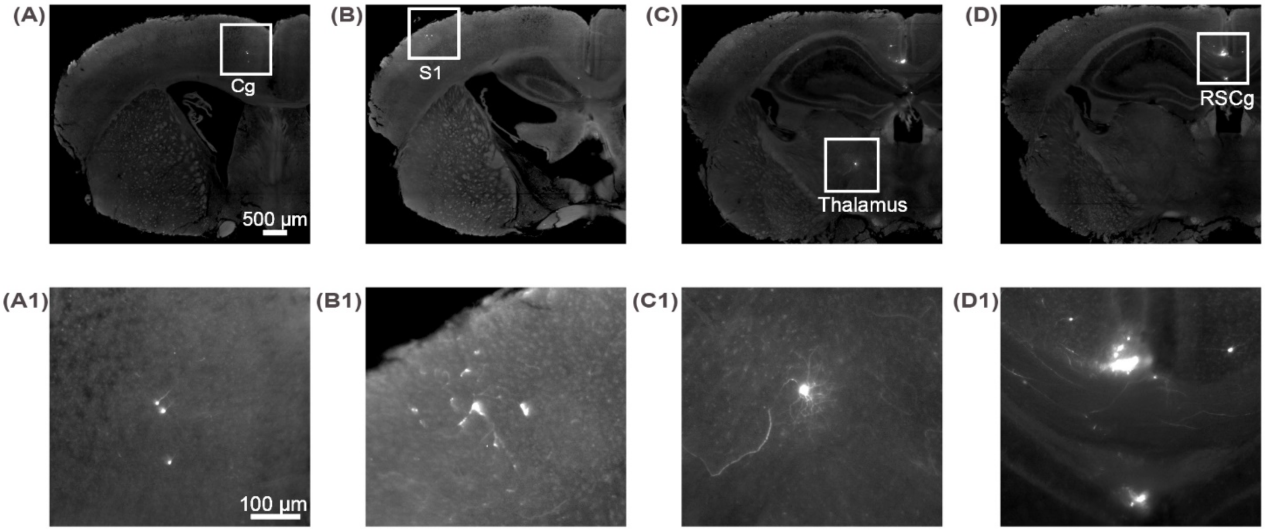
(A)
Cg
500 µm
(B)
S1
(C)
Thalamus
(D)
RSCg
(A1)
100 µm
(B1)
(C1)
(D1)

Supplementary Figure 5. Individual optical slices show clearly labeled input cells from various regions. (A-D) Single optical planes showing inputs from cingulate cortex (Cg) (A), somatosensory area (S1) (B), thalamus (C), and RSCg (D). A-D share same the scale bar. **(A1-D1)** Zoom-in of A-D. A1-D1 share the same scale bar.

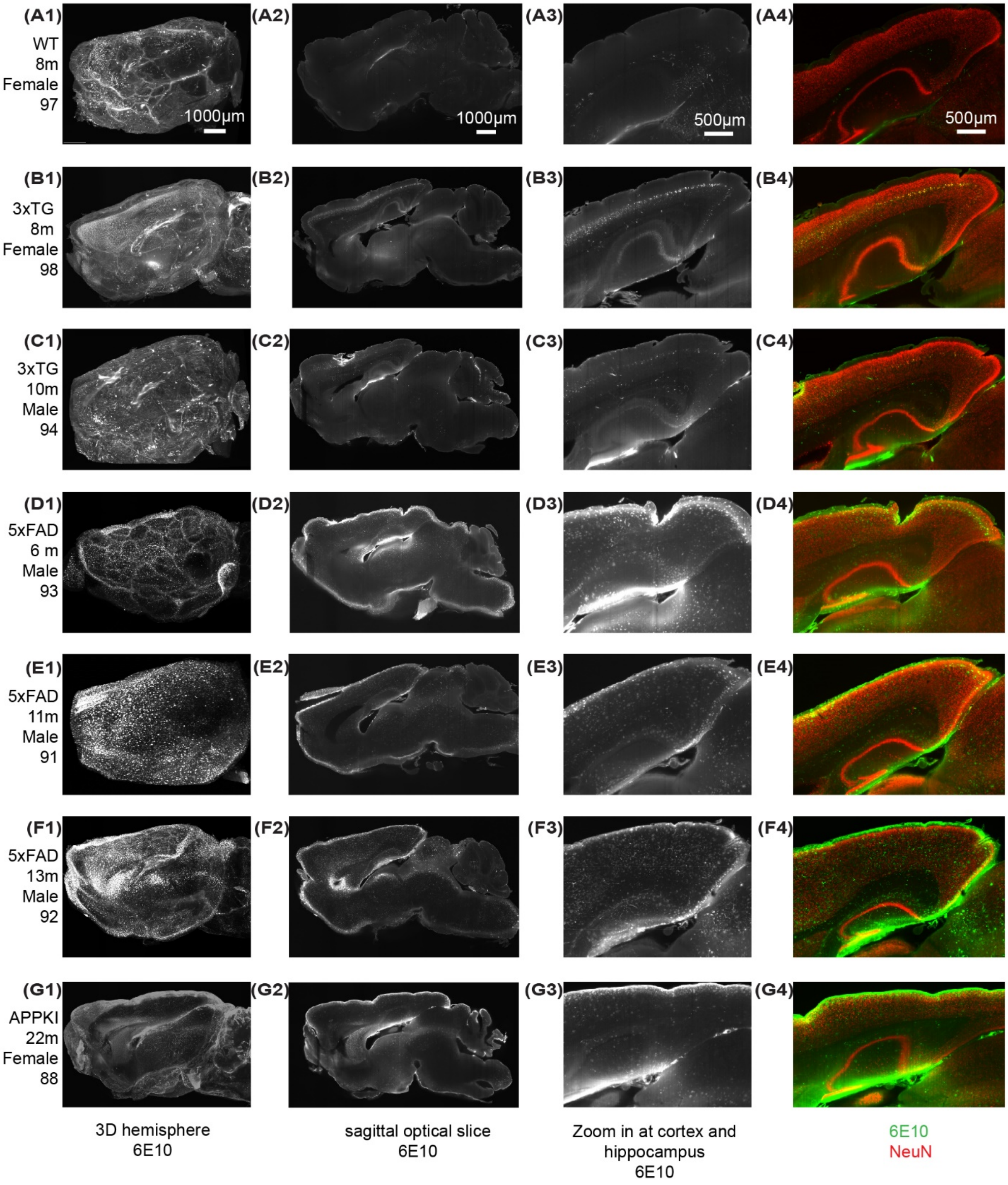
(A1)
WT
8m
Female
97
1000µm
(A2)
1000µm
(A3)
500µm
(A4)
500µm
(B1)
3xTG
8m
Female
98
(B2)
(B3)
(B4)
(C1)
3xTG
10m
Male
94
(C2)
(C3)
(C4)
(D1)
5xFAD
6 m
Male
93
(D2)
(D3)
(D4)
(E1)
5xFAD
11m
Male
91
(E2)
(E3)
(E4)
(F1)
5xFAD
13m
Male
92
(F2)
(F3)
(F4)
(G1)
APPKI
22m
Female
88
(G2)
(G3)
(G4)
3D hemisphere
6E10
sagittal optical slice
6E10
Zoom in at cortex and
hippocampus
6E10
6E10
NeuN

Supplementary Figure 6. iPEGASOS captures the progression of beta-Amyloid accumulation in 3xTG, 5xFAD and APP-KI mice ranging from 6m to 22m of age. (A1-G1) Side views of 3D reconstructed brains illustrate various conditions, with the white signal denoting 6E10 antibody staining for beta-amyloid peptide. A1-G1 shared the same scale bar. The conditions of the mice were labeled on the left. **(A2-G2)** Single sagittal plane views of the respective brains are presented. A2-G2 shared the same scale bar. **(A3-G3)** Zooming in on a section of the cortex and hippocampus (A2-G2) provides a detailed view of the stained beta-amyloid peptide. A3-G3 shared the same scale bar. **(A4-G4)** The same slices as in (A3-G3) are shown here, with the green signal representing 6E10 staining and the red signal corresponding to NeuN staining. A4-G4 shared the same scale bar.

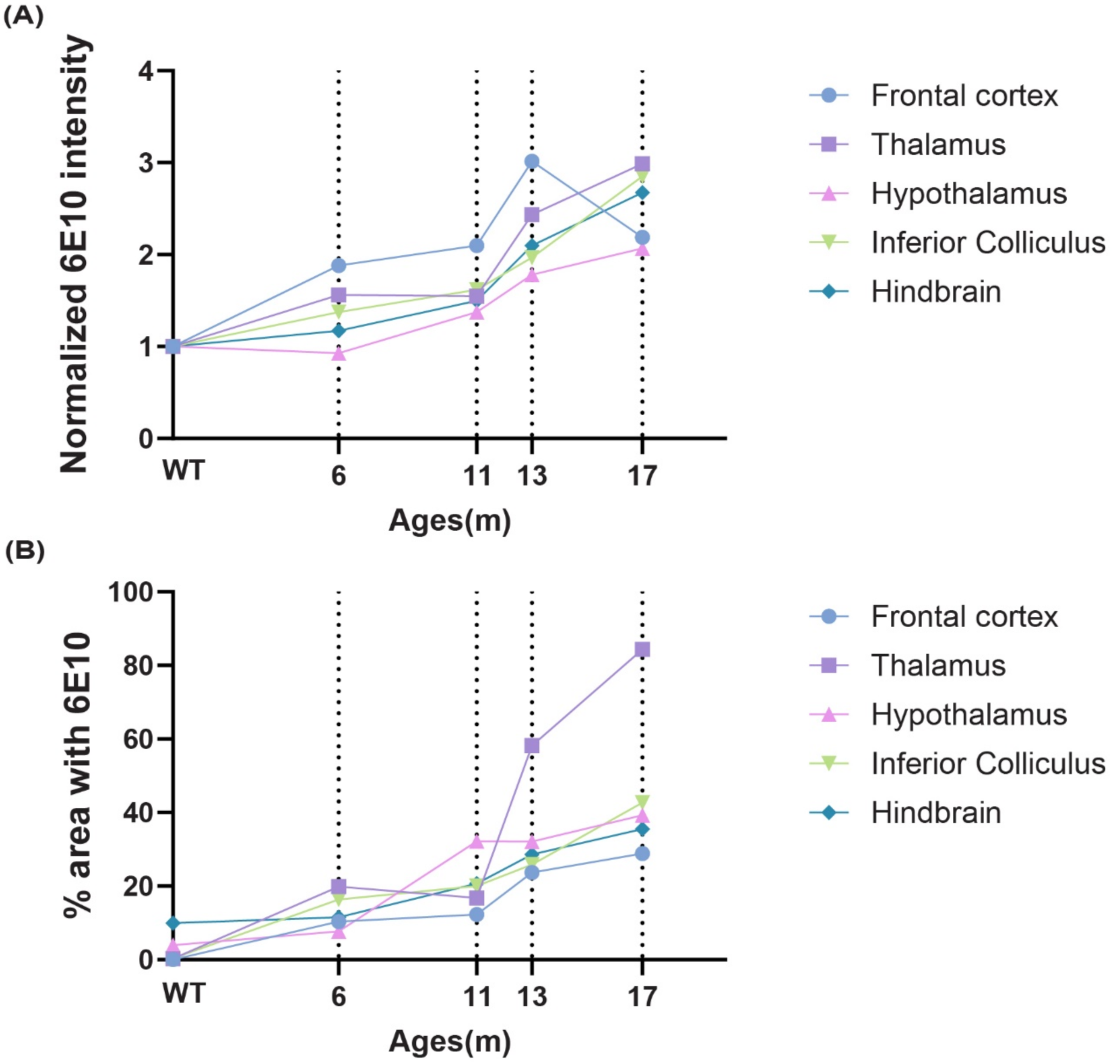
(A)
Normalized 6E10 intensity
0
1
2
3
4
WT
6
11
13
17
Ages(m)
Frontal cortex
Thalamus
Hypothalamus
Inferior Colliculus
Hindbrain
(B)
% area with 6E10
0
20
40
60
80
100
WT
6
11
13
17
Ages(m)
Frontal cortex
Thalamus
Hypothalamus
Inferior Colliculus
Hindbrain

Supplementary Figure 7. Quantification of 6E10 Signal Intensity and Area Percentage with 6E10 Signal in Specific Brain Regions of 5xFAD Mice (A) The signal mean intensity in distinct brain regions (frontal cortex, thalamus, hypothalamus, inferior colliculus, and hindbrain) was quantified and normalized to wild-type mice. An age-dependent increase in 6E10 signal intensity was observed in 5xFAD mice. Example measurements were from one WT and four 5xFAD mice at 4 different ages. **(B)** Assessment of the percentage of area exhibiting 6E10 signal within the delineated brain regions frontal cortex, thalamus, hypothalamus, inferior colliculus, and hindbrain) revealed a higher proportion of aging in 5xFAD mice. Example measurements were from one WT and four 5xFAD mice at 4 different ages.

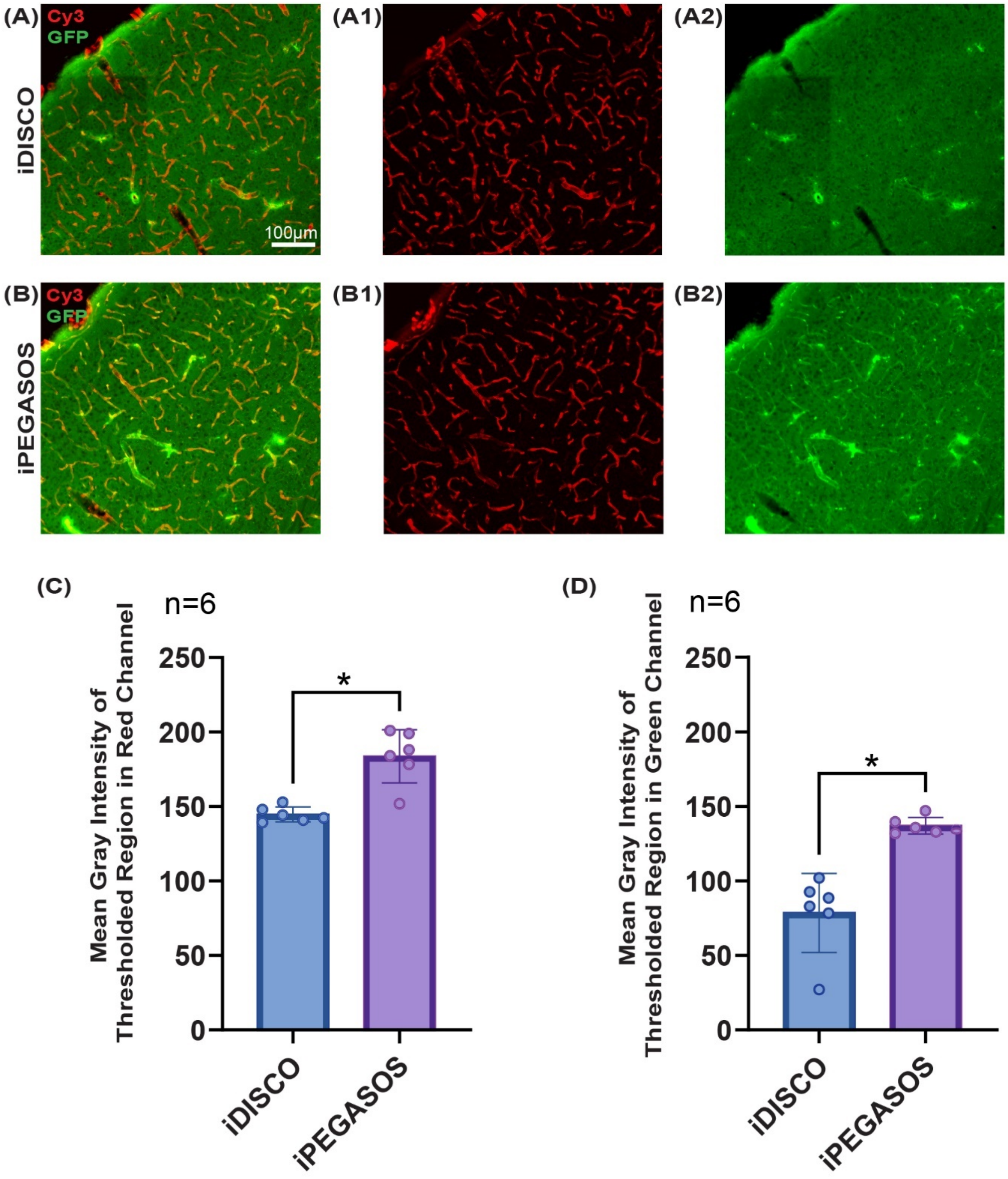
(A)
Cy3
GFP
iDISCO
100μm
(A1)
(A2)
(B)
Cy3
GFP
iPEGASOS
(B1)
(B2)
(C)
n=6
Mean Gray Intensity of Thresholded Region in Red Channel
250
200
150
100
50
0
*
iDISCO
iPEGASOS
(D)
n=6
Mean Gray Intensity of Thresholded Region in Green Channel
250
200
150
100
50
0
*
iDISCO
iPEGASOS

Supplementary Figure 8. Comparative Assessments of Fluorescent Signal Retention in Tie2-GFP Mouse Brain Sections following iDISCO and iPEGASOS. (A) Tie2-GFP mouse brain sections cleared with iDISCO show the immunostained signal in red and the intrinsic GFP expression in green. **(A1)** Displays the immunostained signal. **(A2)** Shows the endogenous GFP signal. **(B)** Tie2-GFP mouse brain sections cleared with iPEGASOS also present the immunostained signal in red, with the GFP expression shown in green. **(B1)** Illustrates the immunostained signal. **(B2)** Presents the endogenous GFP signal. A, A1, A2, B, B1, B2 share the same scale bar. **(C & D)** Blood vessels in the whole section were identified using the ImageJ threshold function to measure the mean gray intensity for both the immunostained and endogenous fluorescence signals across both methods. N=6. Wilcoxon matched-pairs signed rank test to determine significance between the two datasets. **(C)** Reports the measured immunostained signal in red (P value = 0.0312), and **(D)** indicates the endogenous GFP signal in green for samples processed by both iDISCO and iPEGASOS, P value = 0.0312.
