# Supplementary material for: Immunolabeling-compatible PEGASOS tissue clearing for high-resolution whole mouse brain imaging
Source: Front Neural Circuits. 2024 Apr 17;18:1345692. doi: 10.3389/fncir.2024.1345692 (PMC11061518; doi:10.3389/fncir.2024.1345692)
Supplement: Supplementary file 3 [file Data_Sheet_3.pdf]

Data table

Data for Supplementary Figure 2

Sample ID	area	mean	Min	Max
iPEGASOS #1	1844	121.532	29	170
iPEGASOS #2	1844	89.814	23	170
iPEGASOS #3	1844	99.791	32	170
iPEGASOS #4	1844	69.896	27	158
PEGASOS #1	1844	28.58	23	37
PEGASOS #2	1844	23.028	19	170
PEGASOS #3	1844	18.299	16	21
PEGASOS #4	1844	23.049	20	29

Data for Supplementary Figure 4

	N129		N130	
Cell ID	green channel Mean Intensity	cy5 channel Mean Intensity	green channel Mean Intensity	cy5 channel Mean Intensity
1	46.333	225.104	39.227	9.068
2	44.5	101.765	45.568	9.318
3	49.875	160.042	41.795	8.977
4	47.25	80.757	40.75	8.977
5	41.375	109.161	40.337	8.967
6	40.375	189.375	46.023	9.705
7	40.5	66.408	50.136	9.659
8	36.875	64.945	51.432	9.159
9	40	228.177	46.705	9.023
10	43.25	212.188	39.159	9.091
11	45.125	198.681	71.205	9.318
12	47.778	238.108	44.545	9.227
13	42.556	234.738	41.455	8.955
14	46.75	241.609	44.432	9.227
15	40.667	172.24	38.909	8.636
16	40.583	121.036	41.864	8.818
17	42.417	100.014	34	9
18	48.583	205.896	35.164	8.703
19	45.083	130.114	36.045	8.25
20	45.667	141.63	50.659	9.159
21	44.417	204.05	40.5	9.625
22	43.571	199.6	39.159	9.091
23	47.619	235.947	42.889	9.25
24	42.75	202.929	30	9
25	47.625	169.217	34.886	8.932
26	45.875	240.912	33.523	8.977
27	53	241.275	32.432	8.977
28	52.188	238.047	32.705	8.795
29	51.286	244.2	30.159	8.432
30	52.905	248.286	32.955	9.023
31	35.333	113.03	30.636	9.341
32	36.524	145.792	32.364	9.091
33	36.952	59.691	35	10
34	39.81	66.65	33.273	9.045
35	45.571	115.75	34.705	9.091
36	53.429	146.5	33.886	9.409
37	52.286	116.636	36.636	9.75
38	52.762	115.791	37.477	9.25

39	53.524	161.534	38.636	9.227
40	53.476	116.963	41.5	9.205
41	56.095	250.5	39.091	9.818
42	48.381	141.714	40.614	9.5
43	50	107.654	35.227	9.159
44	48.238	252.333	35	10
45	48.154	60.154	39.5	9.029
46	46	70.192	40.618	9.059
47	44.231	70.75	40.295	8.955
48	42.231	56.556	42.636	8.955
49	42.615	71.125	41	8.909
50	40.615	238.75	36.295	8.614
51	40.846	80.452	34.318	8.795
52	40.769	238.548	34.136	8.568
53	42.269	249.161	33	8.205
54	38.615	218.281	31.795	8.205
55	36.346	183.719	34.25	8.636
56	35.538	113.27	34.633	8.664
57	35.846	129.781	35.164	8.703
58	35.269	95.038	35.955	8.523
59	36.115	81.108	35.773	8.295
60	35.654	106.844	37.568	8.727
61	39.346	104.781	36.682	8.727
62	34.5	121	34	9
63	33.808	128.529	34.591	8.864
64	31.654	142.351	35.5	8.727
65	30.577	154.5	32.409	8.864
66	30.923	140.622	30.295	9.136
67	28.808	211.25	29.023	9.182
68	28	180.083	28.909	9.205
69	29.038	103.531	28.909	9.409
70	30.731	114.333	29.318	8.917
71	28.192	116.462	28.523	9.318
72	26.269	133.938	30	9
73	25.5	60.971	29.591	9.114
74	25.808	72.719	29.318	8.917
75	25.462	77.541	32.455	9.386
76	32	62.892	30	9.159
77	31.115	93.118	31.045	9.205
78	32.462	206.429	30.773	9.432
79	32.538	119.324	31.341	9.318
80	31.962	186.581	30.273	9.386
81	29.654	200.038	30.636	9.341

82	28.692	108	54.938	9.062
83	28.75	96.923	50.136	9.659
84	28.844	84.469	52.136	9.591
85	28.125	120.188	53.091	9.136
86	26.156	98.647	54.25	9.068
87	26.719	141.773	52.591	9.159
88	26.531	130.941	57.909	9.159
89	26.625	103.324	63.205	9.182
90	27.969	195.824	63.273	8.932
91	27.219	214.857	66.227	9.273
92	28.062	60.062	73.568	9.25
93	27.719	188.73	71.318	9.091
94	27.688	107.906	75.773	9.477
95	26.844	87.85	72.068	9.25
96	26.692	86.192	71.977	9.182
97	27.692	70.133	68.318	9.182
98	29.346	101.462	70.841	9.364
99	28.462	118.231	65.977	9.227
100	28.615	59.19	71.205	9.318

Data for Supplementary Figure 8

iDISCO	red		green		iPEGASOS	Red		Green	
sample	Area	Mean	Area	Mean	sample	Area	Mean	Area	Mean
A1_PT1_L0	8389041	139.379	8389041	27.114	A2_PT2_L0	7481347	151.871	7481347	135.02
A1_PT1_L1	9999677	142.365	9999677	101.88	A2_PT1_L1	8889891	199.049	8889891	139.575
A1_PT1_L2	10761102	140.923	10761102	82.86	A2_PT2_L3	10368042	178.353	10368042	135.661
A1_PT2_L0	7908918	144.171	7908918	88.584	A2_PT1_L0	7305913	201.004	7305913	131.8
A1_PT2_L1	8959419	148.005	8959419	78.29	A2_PT2_L1	8750882	183.935	8750882	147.039
A1_PT2_L2	10051799	152.985	10051799	92.634	A2_PT2_L2	9852339	188.033	9852339	132.928
